# Multicomplex Pharmacophore Modeling of Estrogen Receptors Suggests the Probable Repurposing of Procaterol as an Antiproliferative Agent Against Breast Cancer Cells

**DOI:** 10.3390/ijms27010463

**Published:** 2026-01-01

**Authors:** Luis Heriberto Vazquez-Mendoza, Humberto L. Mendoza-Figueroa, Nadia Judith Jacobo-Herrera, Norbert Bakalara, Daphne Edith González-Juárez, José Correa-Basurto, Juan Benjamín García-Vázquez

**Affiliations:** 1 Laboratorio de Diseño y Desarrollo de Nuevos Fármacos e Innovación Biotecnológica (Laboratory for the Design and Development of New Drugs and Biotechnological Innovation), Escuela Superior de Medicina del Instituto Politécnico Nacional (ESM-IPN), Plan de San Luis y Salvador Díaz Mirón S/N, Casco de Santo Tomás, Mexico City 11340, Mexico; lvazquezm1903@alumno.ipn.mx (L.H.V.-M.); jcorreab@ipn.mx (J.C.-B.); 2 Unidad de Bioquímica, Instituto Nacional de Ciencias Médicas y Nutrición Salvador Zubirán, Mexico City 14080, Mexico; nadia.jacoboh@incmnsz.mx; 3 Ecole Nationale Supérieure de Technologie des Biomolécules de Bordeaux (ENSTBB), Université de Bordeaux, CNRS, Bordeaux INP, CBMN, UMR 5248, F-33600 Pessac, France; norbert.bakalara@enstbb.fr; 4 División de Biología Molecular, Instituto Potosino de Investigación Científica y Tecnológica (DBM-IPICYT), y Secretaría de Ciencia, Humanidades, Tecnología e Innovación (SECIHTI), San Luis Potosí 78216, Mexico; daphne.gonzalez@ipicyt.edu.mx; 5 Investigadoras e Investigadores por México SECIHTI -Sección de Estudios de Posgrado e Investigación de la Escuela Superior de Medicina, Instituto Politécnico Nacional, Plan de San Luis y Salvador Díaz Mirón S/N, Casco de Santo Tomás, Mexico City 11340, Mexico

**Keywords:** pharmacophore modeling, drug repurposing, estrogen receptor beta, estrogen receptor alpha, breast cancer, procaterol

## Abstract

Breast cancer (BC) is a malignant tumor that develops in the mammary gland due to uncontrolled cell proliferation. Estrogen receptor (ER) signaling, mediated by 17β-estradiol (E2), plays a crucial role in regulating cell proliferation, differentiation, and survival. Specifically, the binding of E2 to the estrogen receptor alpha (ERα) increases cell proliferation. Conversely, selective estrogen receptor beta (ERβ) agonists inhibit cancer cell proliferation by suppressing the expression of oncogenes, making ERβ an important therapeutic target. Given the urgent need for targeted and effective therapies for BC, we implemented a strategy based on multicomplex pharmacophores modeling of ERβ (MPMERβ) and ERα (MPMERα), performing a virtual cross-screening of databases of clinically approved and experimental drugs to identify those with high affinity and stereoelectronic complementarity with the ERβ agonist pharmacophore hypothesis. The implementation of a chemoinformatic strategy enabled the identification of Sobetirome, Labetalol, and Procaterol as molecular hits on the ERβ pharmacophore map. Procaterol showed the most significant antiproliferative activity in vitro assays, with IC_50_ values of 21.26 and 36.10 µM in MCF-7 and MDA-MB-231, respectively. It is imperative to note that these findings require experimental validation of the ERβ activation pathways to strengthen the possible therapeutic repurposing of the drugs selected through our in silico approach. Finally, this strategy not only facilitates drug repurposing under in silico simulation but also provides valuable information for the rational design of new drugs against BC.

## 1. Introduction

Breast cancer (BC) is a malignant tumor developed in the mammary gland because of uncontrolled cell growth and proliferation [1]. It is currently the most prevalent cancer worldwide and the fifth leading cause of death from cancer in general; however, it has the highest mortality rate from cancer in women [2]. According to statistics, the WHO estimates that by 2040 the incidence of BC will rise by 34% and mortality by 52% [3]. Regardless of advances in diagnosis and the application of improved therapies, BC-related morbidity, and mortality are constantly increasing. A high percentage of deaths related to this type of cancer occurs due to metastatic phenomena; according to clinical observations the spread of BC cells predominantly to the bones, lungs, liver, brain, and lymph nodes has been reported [4]. Initial drug therapies, such as those aimed at inhibiting hormone receptors or growth factor kinases, can become ineffective after recurrence and lead to the spread of cancer cells due to the development of chemotherapy resistance. This situation is further complicated in triple-negative breast cancer (TNBC) because traditional FDA-approved treatments are often ineffective. One of the firs line drugs is tamoxifen, an ERα antagonist. However, despite its effectiveness, it has been associated with some adverse effects, such as the development of mutations in tumor suppressor genes and antiestrogen resistance [5]. Currently, two types of nuclear estrogen receptors are known: the ERα and estrogen receptor beta (ERβ). These receptors are transcription factors that regulate various physiological processes related to cell growth, development, and differentiation. They are primarily activated by 17β-estradiol (E2), triggering rapid genomic signaling events that vary depending on the receptor stimulated. Therefore, agonist or antagonist effects on ERs can lead to the design of strategies for developing drugs with specific pharmacological activities [6].

Therapeutic approaches emphasize the importance of understanding the physiological roles of ERα and ERβ in tissues, as well as their mechanistic functionality, to identify effective treatments and minimize side effects. For instance, the binding of E2 to ERα enhances cell proliferation [7]. In contrast, selective ERβ agonists inhibit BC cell proliferation by suppressing the expression of oncogenes such as c-myc, decreasing cyclin D1 expression, and increasing apoptosis [8]. Clinically, increased ERβ expression is associated with a favorable prognosis and improved survival in patients with TNBC [9]. Furthermore, selective ERβ activation slowed the proliferation of TNBC cells sensitive and resistant to traditional chemotherapy. This suggests that ERβ agonists may be active even in cases of advanced and refractory disease in a subgroup of patients [10]. This highlights the potential of leveraging ERβ expression/activation as a therapeutic approach for managing TNBC.

ERβ exhibits a high degree of homology with ERα and exerts its biological functions through both ligand-dependent and ligand-independent mechanisms. This high homology is localized to its DNA-binding domain (∼95%), allowing it to regulate similar target genes upon ligand activation. In addition to its genomic action, it can participate in cytoplasmic signaling through pathways such as PI3K/Akt and MAPK/ERK. Its ligand-independent activity can be induced by phosphorylation or modulating other intracellular signals [11]. Key differences in the ligand-binding domain (LBD), such as the Met336 and Ile373 residues in ERβ (Leu384 and Met421 residues in ERα), have enabled the development of selective ERβ agonists, although this remains a challenge to date.

Despite advances in the design and development of ERβ agonists, no molecule dedicated to this pharmacological target is currently available for clinical use. For this reason, repurposing estrogen receptor ligands has been suggested as a treatment for ERα-negative malignancies. For example, Raloxifene has recently demonstrated antitumor effects in various ERα-negative cancer models, including TNBC [12], hepatocellular carcinoma [13], pancreatic ductal adenocarcinoma [14], and AR-negative prostate cancer [15], by activating ERβ.

To address this challenge and develop new approaches to ERβ-selective ligands, cheminformatics strategies have been implemented, which have facilitated the identification of new bioactive structural scaffolds and repurposing proposals through virtual screening with pharmacophore mapping [16], docking and molecular dynamics simulations [17,18,19]. Specifically, 3D pharmacophores offer an intuitive and powerful representation of interaction patterns in ligand-protein and protein-protein complexes. Three-dimensional pharmacophore modeling allows for the rationalization of the binding modes of chemically diverse ligands and, subsequently, the rapid and highly efficient virtual screening of large collections of compounds to find so-called hits, i.e., new molecules that meet the pharmacophore characteristics necessary to be potentially active against a specific therapeutic target [20]. Since a pharmacophore model does not represent exact chemical groups, but rather chemical features and their spatial relationships with the amino acids from the therapeutic target, the hits identified are often structurally different, making them useful tools for scaffold hopping, allowing for the exploration of a larger chemical space [21]. Pharmacophore modeling-based virtual screening on has been effectively applied in drug repurposing and in the search for biological activity in a wide variety of molecules. There are several in silico proposals and others with interesting experimental validations targeting cancer [22,23,24], neurodegenerative diseases [25], and infectious diseases [26,27]. Currently, pharmacophore modeling can integrate new deep learning algorithms with relevant experimental data, allowing chemically accurate and biologically relevant pharmacophore characteristics to be defined in *apo*-proteins [28], protein-ligand complexes [29], and protein-protein complexes [30], thereby increasing the likelihood of in vivo efficacy and clinical success. In oncology applications, these models can integrate tumor genomics and signal pathway activation data or immunotherapeutic complexes [31], facilitating the identification of compounds that act precisely on relevant oncogenic targets.

**Figure 1:**
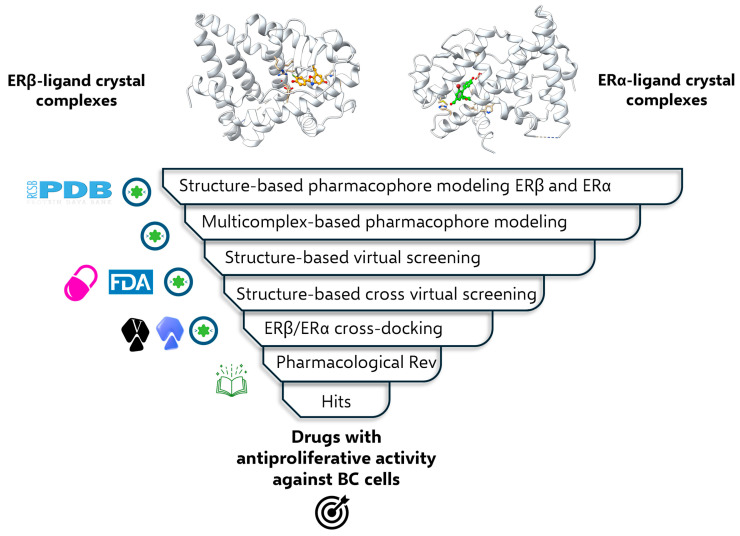
Workflow of the chemoinformatics strategy for identifying drugs with potential for repurposing as anticancer agents. The ligand-protein complexes shown, 1X7B (ERβ) and 4DMA (ERα), are representative of the total crystallographic structures used for pharmacophoric modeling.

In the present study, we implemented a 3D pharmacophore-based virtual screening strategy, complemented with ERβ-targeted molecular docking simulations to identify drugs with structural features and molecular interactions complementary to MPMERβ. This computational approach identified Sobetirome, Labetalol, and Procaterol as molecular hits (Figure 1). Procaterol exhibited an antiproliferative effect in luminal (MCF-7) and triple-negative (MDA-MB-231) BC cell lines at micromolar concentrations, comparable to those of the reference ERβ agonist (Diarylpropionitrile, DPN). Our results provide an exploratory basis for drug repurposing. Therefore, further studies will allow us to explore the antiproliferative signaling pathways related to the modulation of ER activity.

## 2. Results and Discussion

Considering that molecular recognition between a ligand and its therapeutic target determines its pharmacological activity, a structure-based pharmacophore modeling was developed to analyze the physicochemical and electrostatic complementarity of ERβ agonists and ERα ligands, to characterize their stereoelectronic properties, and discriminate the differences in interaction patterns associated with their pharmacological activity and selectivity. This approach enabled the optimization of virtual screening and the selection of molecular hits, establishing an exploratory computational strategy to identify candidates for the repurposing of drugs against BC.

### 2.1. Structure-Based Pharmacophore Modeling

**Figure 2:**
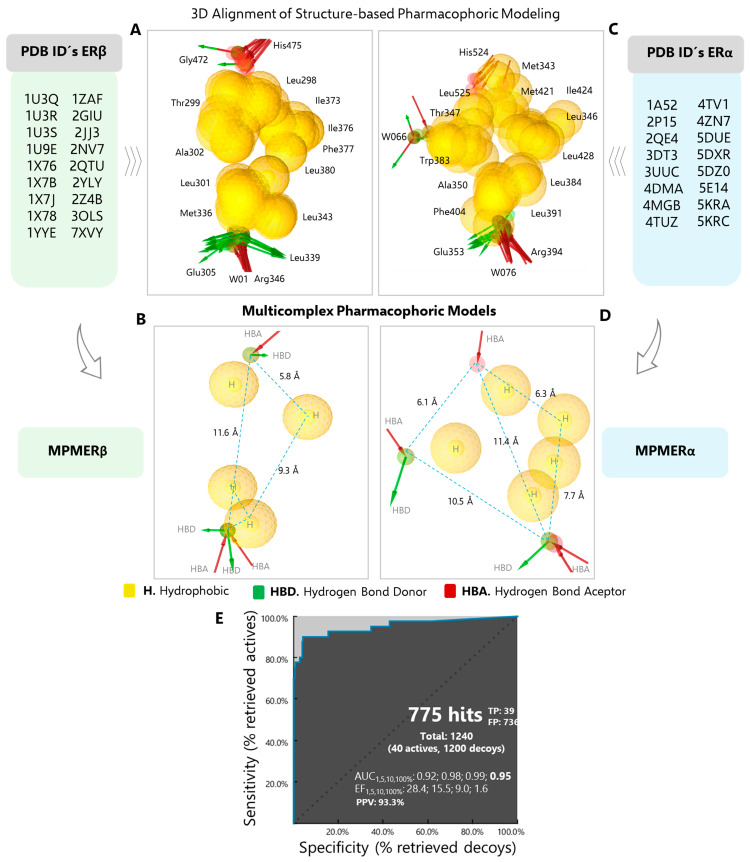
Pharmacophore modeling of ERβ and ERα. (**A**) Alignment of structure-based pharmacophoric models of eighteen agonist- ERβ crystal complexes. (**B**) Pharmacophoric hypothesis obtained from MPMERβ. (**C**) Alignment of structure-based pharmacophoric models of sixteen ERα crystal complexes. (**D**) Pharmacophoric hypothesis obtained from MPMERα. (**E**) Validation of MPMERβ pharmacophoric hypothesis by ROC curve.

In accordance with the structural diversity of the co-crystallized ERβ agonists, pharmacophoric modeling of eighteen complexes was performed (Table S1). The three-dimensional alignment of each model revealed a high degree of coincidence in the positioning of pharmacophoric features. In general, a cluster of central hydrophobic interactions was observed, which was delimited end-to-end by acceptor (Arg346 and His475) and donor (Glu305 and Gly472) hydrogen bond (HBA/HBD) interactions at an average distance of 11.60 Å (Figure 2A,B). The spatial distribution of the hydrophobic interactions generated by the ERβ agonists reveals the presence of a cavity, which is delimited by amino acids that are indispensable for the modulation of pharmacological activity, including Leu298, Met336, Ile373, Ile376, Phe377, and Leu380 [32,33,34]. In contrast, the distribution of hydrophobic interactions generated by ERα ligands demonstrates that the ERα binding site exhibits enhanced conformational flexibility (Figure 2C; individual models are shown in Table S2). It has been demonstrated that the ERα-affinity and selectivity are dependent on the nature of the ligand and its ability to interact with Met343, Ala350, Leu384, Leu391, Phe404, Met421, Ile424, Phe425, Leu428, and Leu525 [35]. The alignment of the pharmacophoric models revealed a high degree of coincidence in the HBD, and HBA interactions generated by the crystal lattice of Glu353, Arg394, and a water molecule, respectively (Figure 2C). These interactions are primarily attributed to the presence of a hydroxyl group (or bioisostere) in the ligand structure and are regarded as being of fundamental importance in molecular recognition [36,37]. Another interesting pharmacophoric feature is the HBA formed by His524, which was predominantly observed in the presence of agonist ligands [38]. In contrast, antagonists yielded an HBD and an additional HBA interaction with Asp351, as well as a water molecule, respectively. These interactions have proven to be indispensable in the design of more potent and selective drugs [39,40,41].

### 2.2. Multicomplex-Based Pharmacophore Modeling and Validation

Structure-based pharmacophore models are typically generated from crystallographic data of apo structures or of a single protein-ligand complex [21]. However, in cases where there is a diversity of ligand binding modes with the same target and pharmacological activity, a pharmacophore based on multicomplexes is the optimal choice. This approach enables the identification of several protein-ligand interaction patterns at the recognition site and the assessment of their importance, which is essential for differentiating between agonist and antagonist effects. The resulting integrative pharmacophoric mapping contains a greater quantity of chemical structural information and enhances the degree of precision in the selection of molecular hits in a virtual screening analysis [42,43].

The MPMERβ was constructed by combining the pharmacophoric characteristics of eighteen crystallographic structures, with ERβ-Genistein, a high-affinity agonist [44], serving as the reference point. The rationale behind this superposition was to transform the coordinates of the receptor, the agonists, and the exclusion volumes into a unified framework. The MPMERβ pharmacophoric hypothesis (Figure 2B) showed a total of ten features; specifically, four coplanar hydrophobic (H) interactions surrounded by Leu298, Thr299, Leu301, Ala302, Met336, Leu339, Leu343, Phe356, Ile373, Ile376, Phe377 and Phe380, spanning a trapezoidal area of 28.9 Å^2^; three HBD interactions with residues Glu305, Leu339 and Gly472 and finally three HBA interactions with residues Arg346, His475 and a water molecule (W01). Conversely, the superposition of sixteen pharmacophoric models of ERα resulted in the MPMERα (Figure 2D). Upon merging the shared features, the resulting model was reduced to eleven, of which five correspond to hydrophobic interactions centrally distributed in an approximate area of 23.0 Å^2^ [45]. Additionally, four HBA were identified, with Arg394, His524, and two-water molecules (W066 and W076). Finally, two HBD interactions were observed with Glu353 and W066. It is noteworthy that the MPMERα contains the interactions essential for affinity, as well as those that determine the agonist and antagonist activity of the ligands with which it was generated [46].

Selective targeting of ligands to ERβ over ERα is a major challenge, given the high sequence identity in the ligand-binding domain and DNA. Nevertheless, several chemically diverse ERβ agonists have been described with activity in the nanomolar range and even greater than 100-fold selectivity over ERα [47,48]. Considering the above, two sets of training ligands, TLERβ and TLERα (Table S3), were created to validate the predictive ability and selectivity of MPMERβ and MPMERα models. Specifically, following the virtual screening of both sets of ligands, the MPMERβ and MPMERα models demonstrated the highest pharmacophore fit scores for those ligands that exhibited a correlation with their reported pharmacological activity (Table S4). The MPMERβ model demonstrated the capacity to fit 23 ERβ agonists on the pharmacophoric features, out of a total of 32 in TLERβ. This was achieved with a pharmacophore fit score higher than those obtained for TLERα set, considering the score threshold of 66.86, preferential for MPMERβ.

This outcome validates and highlights the predictive and preferential capacity of the multicomplex model to filter ERβ agonists despite the structural homology of the receptors and the molecular similarity of the ligands [49,50].

ROC curve analysis was performed using a set of active estrogen receptor modulators (including agonists and antagonists), as well as a database of decoy molecules. These molecules present a challenge to the model because, although they exhibit high structural similarity and physicochemical properties to the active compounds, they lack relevant biological activity [51]. This approach ensures a more rigorous and realistic assessment of the predictive performance of the MPMERβ pharmacophore hypothesis. As illustrated in Figure 2E, the model identified a total of 775 matches, of which 39 were determined to be true positives (estrogenic molecules) and 736 were classified as false positives (decoys). The evaluation of the overall performance of the model, as measured by the area under the curve (AUC), yielded a value of 0.95, reflecting its excellent discriminatory ability to distinguish between active and inactive molecules. Furthermore, analyzing the ROC screening results with a score threshold of 77.94 (pharmacophore fit score) identified twenty-eight active compounds and only two decoys, yielding a positive predictive value of 93.33%, as shown in Table S5. These results suggest that the model can accurately predict pharmacological activity.

### 2.3. Multicomplex Pharmacophore-Based Virtual Screening

Once the pharmacophoric models were validated, virtual screening was performed using the FDA and DrugBank databases (including clinically used, investigational, and experimental drugs). Initially, the protocol for selecting the best candidates involved identifying structural conformers that correlated with the pharmacophoric characteristics that determine the ERβ agonist effect (MPMERβ). At this stage, 592 structures were identified that matched at least five characteristics. Subsequently, a more exhaustive selection criterion was applied to this set, including exclusion volumes, fragment searching, and matching with at least six characteristics. It is important to consider these parameters when the ligand-binding domain of the therapeutic target adopts different conformational states according to its structural plasticity and, particularly, when homology is high and steric effects determine the modulation of the receptor by its ligand [52]. This reduced the number of hits to 27 drugs. To avoid potential structural biases, the MPMERα model was used to filter the top 27 selections. The model allowed the ERβ/ERα selectivity ratio to be calculated, considering the pharmacophore fit score (Table 1). During the screening, the reference molecules were 8-ethyl-6-(3-fluoro-4-hydroxyphenyl)-naphthalen-2-ol (CHEMBL193064) and *S*-Diarylpropionitrile (*S*-DPN) due to their high affinity and selectivity as ERβ agonists. For ERα, the selective antagonist 5-(4-hydroxyphenoxy)-6-(3-hydroxyphenyl)-7-methylnaphthalene-2-ol (GW368) and the selective agonist propyl pyrazole triol (PPT, a positive control for cell proliferation) were filtered out.

Based on the decreasing pharmacophore fit scores in Table 1, three ERβ agonists were identified in DrugBank: DB06875 (WAY-202196), DB06832 (Prinaberel), and DB01645 (Genistein). These agonists, along with the two reference compounds, have experimental estrogenic activity [53]. These results demonstrate the excellent ability of the MPMERβ model to select potentials agonists, including DB07425 (Sobetirome) and DB14008 (Hispidulin). In all cases, ERβ/ERα ratios greater than one were observed. Interestingly, Labetalol, Procaterol, and Fulvestrant also showed a preferential trend for the MPMERβ model. On the other hand, MPMERα successfully identified the ERα reference molecules (GW368 and PPT) with pharmacophore fit values of 105.85 and 87.22, respectively. Bazedoxifene, Raloxifene, and Afimoxifene (DB04468) also emerged from the databases with fit values of 106.45, 96.82, and 86.69, respectively. These data were anticipated, as these drugs are used clinically as non-steroidal ERα selective antagonists against BC [54]. It is important to note that estradiol exhibited similar pharmacophore fit scores in MPMERβ and MPMERα (56.89 and 57.09, respectively), which reflects its low agonist selectivity (the conformational fit to MPMERβ of each identified drug is specified in Table S6). Such finding supported the use of estradiol in the final calculation of the theoretical selectivity index ERβ (TSI ERβ).

### 2.4. Molecular Docking

To complement screening and improve the selection of promising drugs, affinity energy predictions were made for both ERs. The calculation parameters were validated by a self-docking method, obtaining RMSD values of less than 2 Å between the bioactive and theoretical conformation observed in each ER (Table S7). Table 1 summarizes the energy values obtained for each drug on both receptors, while Table S8 condenses details of interactions with amino acid residues. Subtle changes in the ligand-binding domain of ERs that differ only in two amino acid residues, Met336 (ERβ) replaced by Leu384 (ERα) in helix H6 and Ile373 (ERβ) replaced by Met421 (ERα) in helix H8 [55], did not significantly modify the affinity (binding free energy = ΔG values) displayed by Estradiol (−10.74 kcal/mol in ERβ and −9.67 kcal/mol in ERα). This similar behavior was observed in the fit to the multicomplex pharmacophoric models, since only the HBA, HBD and two central hydrophobic entities were aligned in both models. Estradiol adopts a binding mode along the receptor cavity, anchoring through a network of hydrogen bonds with Glu305/353 and Arg346/394 (ERβ and ERα, respectively), but leaving empty sub-pockets. This finding indicates a structurally versatile plasticity of the ligand-binding domain, which is conducive to the molecular recognition of a range of structurally diverse agonists and antagonists. Therefore, hydrophobic interactions and steric hindrance are crucial for the selective modulation of each receptor. In consequence, the conformational flexibility of the ligands is relevant, although in some cases the binding energy is not significantly modified. This statement can be verified using the reference ERβ agonists CHEMBL193064 and *S*-DPN, which exhibited docking energy differences of 1.22 and 0.53 kcal/mol, respectively. However, they presented a much better conformational fit to the ERβ pharmacophoric conditions than the ERα map. Similarly, the reference ERα molecules (GW368 and PPT) exhibited slight variations in binding energies and demonstrated an excellent fit to the MPMERα. Hanson et al. [44] published an experimental example related to these findings. They synthesized potent and selective ERβ agonists (SERBAs) streamlined to the A–C rings of the cyclopentanoperhydrophenanthrene structure. The most potent and selective derivative, ISP358-2, exhibited 750-fold greater selectivity for the ERβ than for the α-isoform and seven other nuclear receptors, with EC_50_ values between 20 and 30 nM in direct binding and cellular assays. This derivative’s potency and selectivity are attributed to the conformational freedom of the cyclohexane ring and the *S* configuration of the -CH_2_OH fragment compared to its conformationally unsaturated analogs. Analyses of affinity and binding mode revealed that ISP358-2 had an energy of −8.0 kcal/mol at ERβ and −7.6 kcal/mol at ERα. It also maintained interactions that determine the agonist effect. These include hydrogen bonds with Glu305 and Gly472, π-π interactions with Phe355 and Phe356, and hydrophobic interactions with Leu298, Ala302, Ile373, and Leu476 [56]. These data correlate with our results (Table 1. Affinity Energy).

**Figure 3:**
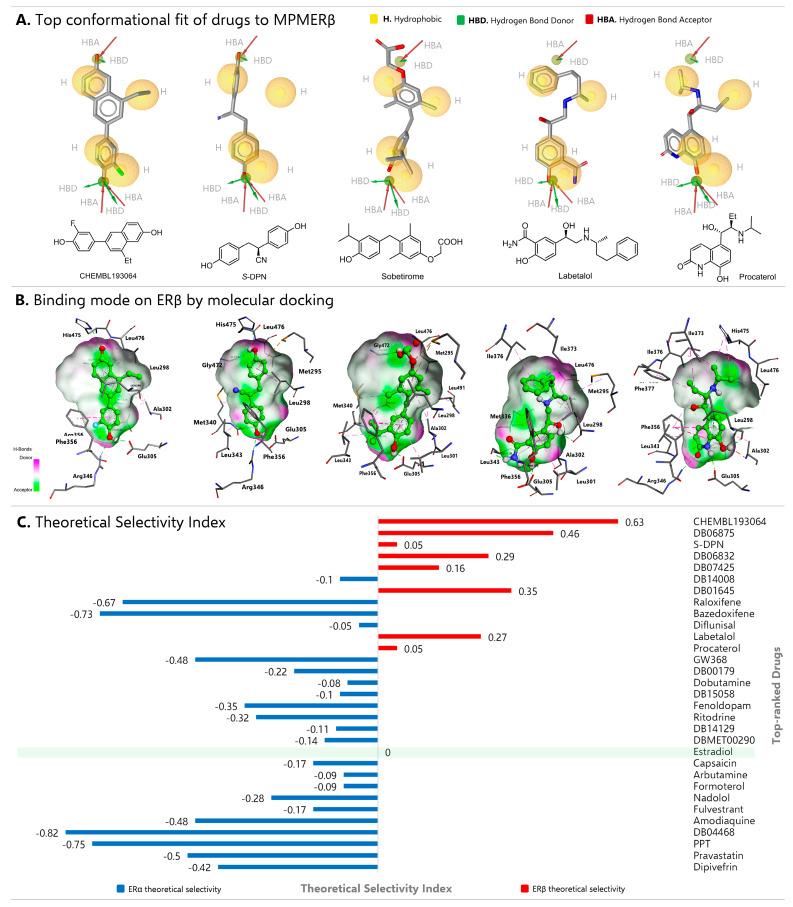
Drugs with the best theoretical selectivity index towards ERβ. (**A**) Structural conformer with the best pharmacophore fit score on MPMERβ; CHEMBL193064, Diarylpropionitrile (S-DPN), Sobetirome, Labetalol, and Procaterol. (**B**) Binding mode, by molecular docking on ERβ. The binding modes are shown with molecular surfaces highlighting the HBAs in green and the HBDs in pink. (**C**) Theoretical selectivity index of drugs identified by virtual screening based on multicomplex pharmacophore modeling. Selective ERβ agonists were identified (red bars), such as CHEMBL193064, DB06875 (ERB-196), S-DPN, DB06832 (Prinaberel) and DB01645 (Genistein), as well as promising drugs for repurposing, such as Sobetirome, Labetalol and Procaterol.

Among the drugs with the highest free energy values on ERβ from docking simulations, DB06875 (ERB-196), DB06832 (Prinaberel), and DB01645 (Genistein) were identified. Despite not having formal approval for clinical use, these three drugs have been experimentally characterized for their high ERβ selectivity [57,58]. According to our workflow, we also managed to identify a drug currently in clinical investigation (Sobetirome, DB07425) and two FDA-approved drugs, Labetalol and Procaterol. Sobetirome exhibited a preferential binding energy for ERβ (−9.70 kcal/mol) and maintained HBA interactions with Glu305 and Gly472, π-π interactions with Phe356, and hydrophobic interactions with Met295, Leu298, Leu301, Ala302, Leu476, and Leu491, which are the amino acids that make up the selectivity sub-pocket (Figure 3B). Labetalol maintained only one HBA interaction with Glu305, presented one π-π interaction with Phe356, and hydrophobic interactions with the selectivity sub-pocket, as well as with residues Met336, Leu343, Ile373, or Ile376, with a binding free energy of −9.51 kcal/mol on ERβ. Procaterol exhibited a binding ΔG of −8.71 kcal/mol, like the agonist DPN. The 1,2-dihydroquinolin-2-one fragment favored HBA interactions with the backbone of Ala302 and the acid group of Glu305, as well as HBD interactions with Arg346. Due to its binding mode (Figure 3B), Procaterol exhibited π-π interactions with Phe356 and π-alkyl interactions with Phe377 and His475. It also maintained hydrophobic interactions with Leu298, Ile376, and Leu476. A detailed description of the interactions with amino acid residues for all selected drugs is presented in Table S7.

The theoretical selectivity index was calculated from these data, and the normalized results (with the values obtained for estradiol) are shown in Figure 3C. Among the eight compounds with the best ERβ selectivity, Sobetirome, Labetalol, and Procaterol stand out due to their lack of estrogen receptor-related therapeutic indications. Specifically, Sobetirome selectively binds to the major hepatic form of the thyroid hormone receptor (TRβ1), clinically exerting its therapeutic activity by stimulating hepatic pathways, which confer antilipidemic and antiatherosclerotic properties [59]. Furthermore, it has been shown to have an important neuroprotective effect in multiple system atrophy, a condition observed in neurodegenerative diseases, since it has been proven to increase the expression of myelinating proteins and the length of the myelinated segment in primary murine oligodendrocytes with overexpression of α-synuclein [60]. The functional remyelination effect was previously demonstrated with selective ERβ agonists such as DPN and phenyl-2*H*-indazole derivatives [61]. Therefore, presumably, Sobetirome exerts its neuroprotective effect by modulating estrogen receptors and acting on cholesterol homeostasis, which has been proposed as a therapeutic target in multiple sclerosis [62]. Based on this information and our screening results, Sobetirome is a promising molecular target for evaluating its suppressive activity in BC. On the other hand, Labetalol, a nonspecific α/β adrenergic antagonist used to treat hypertension, angina, and sympathetic hyperactivity syndrome, has not been investigated in cancer, unlike Propranolol (a β1 selective blocker) [63], so it is important to evaluate its antiproliferative activity in BC cells. Procaterol, meanwhile, is a potent bronchodilator that acts as a β2-adrenergic receptor agonist. Repositioning studies have revealed its ability to significantly restrict CDK12 kinase activity and inhibit the proliferation of human gastric cancer cells [64].

### 2.5. Antiproliferative Activity Assays on BC Cell Lines

**Figure 4:**
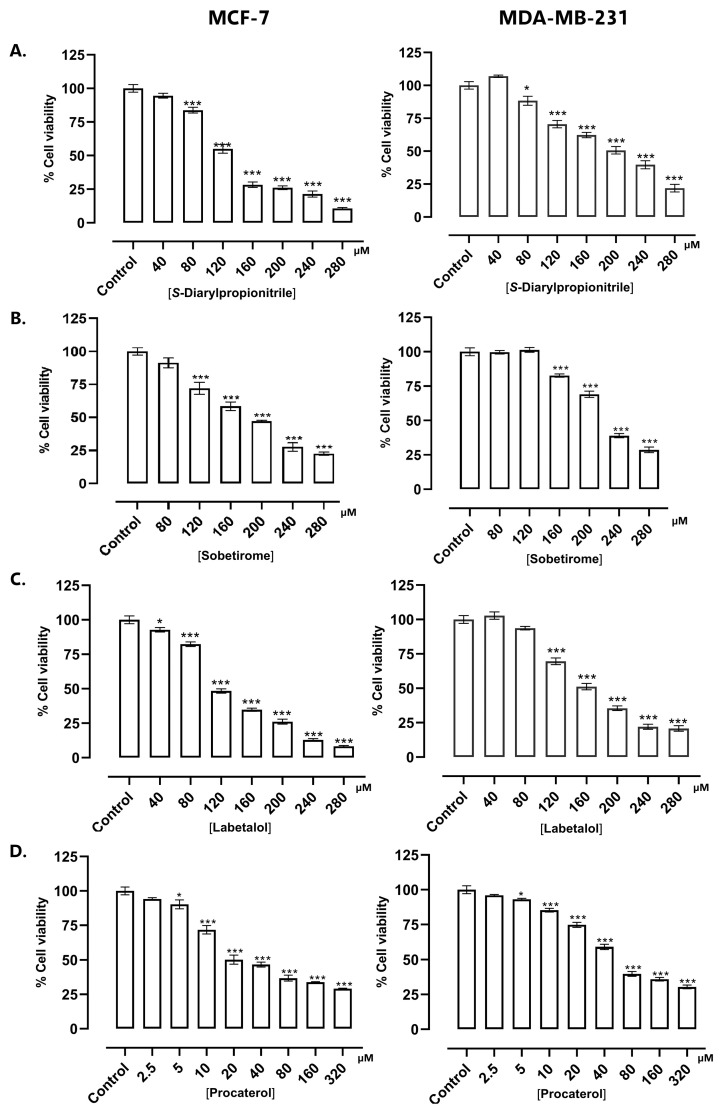
Antiproliferative activity of the best-ranked drugs, according to MPMERβ-based virtual screening, on MCF-7 and MDA-MB-231 cell lines. (**A**) *S*-Diarylpropionitrile (S-DPN); (**B**) Sobetirome; (**C**) Labetalol; and (**D**) Procaterol. The MTT assay was used to determine the percentage of cell proliferation. All conditions were evaluated with six technical replicates in three independent biological experiments. Data is presented as mean ± standard error (SE) following one-way analysis of variance (ANOVA) with Dunnett’s post hoc test. * *p* < 0.05, and *** *p* < 0.001 compared to control.

Based on data obtained from virtual screening of multicomplex pharmacophoric models, molecular docking, the theoretical selectivity index, and current pharmacological information Sobetirome, Labetalol, and Procaterol were selected as promising candidates to evaluate their antiproliferative effects on BC cells. The impact of the drugs on cell growth inhibition was assessed in MCF-7 (luminal) and MDA-MB-231 (TNBC) cell lines using a standardized MTT assay. The cell lines were selected based on their estrogen receptor expression. MCF-7 cells express high levels of ERα, which is essential for their proliferation and sensitivity to estradiol; however, ERβ expression is low. In contrast, TNBC cells lack ERα expression. However, they can express ERβ and the G protein-coupled estrogen receptor (GPER-1) in low proportions, which modulates the response to estrogen [65]. Given that ERα is primarily responsible for the initiation and progression of BC, therapeutic alternatives are based on molecules that inhibit its activity. To date, three main strategies have been identified: the use of aromatase inhibitors, selective estrogen receptor modulators, and selective antagonists [36]. Conversely, ERβ activation has been linked to antiproliferative effects in disease progression and the promotion of positive gene self-regulation [66,67]. From this perspective, the effect of the selective ERβ agonist, S-DPN, was evaluated at six concentrations (40, 80, 120, 160, 200, 240 and 280 µM) at 48 h. These concentrations correspond to the range in which a biologically significant effect on cell viability was observed during a pre-evaluation using logarithmic concentrations from 0.01 µM to 310 µM. *S*-DPN had a concentration-dependent behavior, with an IC_50_ value of 122.50 µM and 186.85 µM in MCF-7 and MDA-MB-231, respectively (Figure 4A). The antiproliferative effect observed at relatively high concentrations is related to the ERα/ERβ expression ratio in each cell line [68]. When both receptors are expressed at similar levels, ERβ activation decreases cell growth and the number of cell cycle components involved in proliferation [69].

Indeed, studies have shown that when ERα and ERβ are co-transfected into ER-negative cells, ERβ inhibits ERα’s transcriptional activity and reduces the cells sensitivity to E2. ERβ also reduces ERα mRNA and protein levels in MCF-7 cells, thereby indirectly influencing their function [70]. Overexpression of ERβ in MCF-7 cells can inhibit the regulation of a subset of genes involved in DNA replication, cell cycle regulation, and proliferation by ERα, as well as cell proliferation in response to E2, by increasing the expression of antiproliferative genes such as p21^Cip1^ and p27^Kip1^ [71]. On the other hand, the direct antiproliferative activity of ERβ agonists on MDA-MB-231 cells is inconsistent. Some authors mention that agonists such as Prinaberel, WAY-200070, 3β-Adiol, or Liquiritigenin do not significantly affect the proliferation of this cell line at concentrations <10 μM but do have a good effect in reducing invasion [72]. In contrast, a study evaluating the re-expression of the ERα and ERβ in MDA-MB-231 using epigenetic drugs (Decitabine and Vorinostat) and ERβ agonists (DPN) inhibited cell growth with an IC_50_ of 0.093 μM at 48 h and increased ERβ expression, especially when the analysis was performed with triple combination therapy scheme (DNA methyltransferase inhibitor/Histone deacetylase inhibitor/ERβ agonist) [73]. Although the observed concentrations differ from those reported, it is evident that a higher concentration of DPN is required to inhibit the growth of ER-negative cells. However, a non-receptor-related effect due to high concentrations cannot be ruled out, as it was previously reported that DPN had an antiproliferative effect on melanoma cells (B16F10) at >100 μM after 48 h, an effect that did not depend on ERβ activation [74].

The hits identified in the virtual screening showed a concentration-dependent inhibitory effect on cell viability. Sobetirome obtained an IC_50_ of 170.50 μM and 206.00 in MCF-7 and MDA-MB-231, respectively. Labetalol showed an effect with an IC_50_ of 127.50 μM in MCF-7 and 146.30 μM in MDA-MB-231. Subject to elucidation of the specific molecular mechanisms underlying the observed effect, a cytotoxic concentration-dependent impact cannot be ruled out. Procaterol had the best activity in both cell lines, obtaining an IC_50_ of 21.26 μM in MCF-7 and an IC_50_ of 36.10 μM in MDA-MB-231 cells (Table 2).

The antiproliferative activity of Sobetirome is promising. According to our in silico approach, structurally, it presents the conditions required to maintain the appropriate number of pharmacophoric features consistent with MPMERβ. Sobetirome is a thyromimetic drug that acts on the thyroid receptor TRβ1, stimulating the hepatic pathways that reduce cholesterol. However, it has anticancer effects in anaplastic thyroid carcinoma with a history of mutations, reducing tumor phenotypes and cancer cell populations. Furthermore, in xenograft trials, it inhibited tumor growth on its own and was as effective as Sorafenib, an FDA-approved antineoplastic drug for the treatment of unresectable hepatocellular carcinoma and advanced renal cell carcinoma [75]. Additionally, TRβ1 activation yields tumor-suppressing functions due to its ability to inhibit tumor initiation, progression, and metastasis [76]. Retrospective analyses suggest that the expression of THRβ is associated with a lower risk of BC recurrence. Therefore, further investigation of the repurposing of THRβ agonists as anticancer agents is warranted [77].

Labetalol, an α, β-adrenergic antagonist, exhibited antiproliferative activity at concentrations above 120 μM in both BC cell lines (Figure 4C). The effect observed at these concentrations can be analyzed from a structure-activity relationship perspective. Labetalol has structural fragments that favor molecular recognition by ERβ, specifically the o-hydroxybenzylamide group, which generates hydrogen bond interactions with key amino acids, and exhibits conformational flexibility with the phenylbutyl fragment. This flexibility allows Labetalol to adopt a binding mode that encompasses the hydrophobic sub-pockets that cover ERβ agonists. Experimental evidence has demonstrated that α-blockers and β-blockers have the capacity to reverse the proliferation of MCF-7 and MDA-MB-23 cells associated with catecholamine stimulation. This suggests a relationship with the expression of adrenergic receptors (AR) in basal cell carcinoma cells [78]. For instance, Propranolol, a non-selective β-blocker, has been demonstrated to inhibit BC cell proliferation in various cell lines (e.g., MDA-MB-231 with an EC_50_ = 78 μM, or SK-BR-3 with an EC_50_ = 18 μM) by decreasing Ki67 protein expression, reducing the phosphorylation of mitogenic signaling regulators, and increasing the phosphorylation of cell survival/apoptosis regulators, such as AKT [79]. Interestingly, these same signaling pathways have been modulated when BC cells are treated with ERβ agonists [80,81]. Based on this background and extensive retrospective evidence, there is a high probability that α- and β-adrenergic blockers can be repositioned, either as monotherapy or in combination with other therapeutic agents, for the treatment of BC [82,83,84].

Procaterol demonstrated the most significant antiproliferative activity against BC cells. The IC_50_ values were consistent with the ERα/ERβ ratio of each cell line, showing a stronger effect in MCF-7 cells than in MDA-MB-231 cells. Given its classification as a β-adrenergic agonist, the potential cytotoxic effect of procaterol may be associated with the activation of additional receptors involved in oncogenic processes [85]. A particularly salient finding is that Procaterol exerts a pronounced inhibitory effect on cell viability and colony formation in a range of cancer cell lines, including those derived from gastric cancer, colon cancer, lung cancer, and squamous cell carcinoma of the esophagus. This effect is attributed to the suppression of CDK12. In addition, in vivo, it demonstrated substantial suppression of tumor growth in various patient-derived gastric tumor xenografts [52]. In parallel, ERβ agonists block cell proliferation, migration, and colony formation, and induce apoptosis and cell cycle arrest in the S and/or G2/M phases in ER-positive BC cell lines [86,87]. Consequently, further experimental validations are necessary to establish the relationship between Procaterol and cell cycle arrest via the activation of ERβ. Additionally, sufficient preclinical studies must be conducted to accurately determine an effective and safe dosage for future anticancer applications in humans.

In this study, the focus was on drugs that propensity for ERβ affinity, considering the prevailing significance of this receptor as a viable pharmacological target. However, the cross-screening approach facilitated the identification of drugs with high affinity for ERα, which, surprisingly, have already demonstrated anticancer activity. Among them, we highlight DB00179 (Masoprocol), which inhibits the activation of two receptor tyrosine kinases (RTKs), the insulin-like growth factor receptor (IGF-1R) and the c-erbB2/HER2/neu receptor, leading to a fall in the proliferation of BC cell populations [88]. Dopamine D1 receptor agonists, such as Fenoldopam, significantly reduce lung metastasis in the 4T1 BC model [89]. The compound DB14129 (Macelignan) has been evaluated in MCF-7 cells. It has been postulated as a novel, natural activator of AMP-activated protein kinase (AMPK), with potential preventive and therapeutic effects against cancer. Studies have demonstrated its apoptotic effects in solid tumors from 4T1 breast carcinoma-bearing mice and in HCT116 colorectal cancer cells in vitro [90]. Capsaicin exhibits antiproliferative properties against both MCF-7 (IC_50_ = 167.90 μM after 48 h) and MDA-MB-231 (IC_50_ = 201.47 μM after 48 h) cells [91]. Statins (such as Pravastatin) can exert anticancer effects through multiple mechanisms, including the mitochondrial apoptosis pathway, the LKB1-AMPK-p38MAPK-p53-survivin signaling cascade, the modulation of the EGFR/RhoA and IGF-1 signaling pathways, and the regulation of the BMP/SMAD4 signaling pathway [92]. Inspired by this finding, we decided to evaluate Amodiaquine to validate our assay according to our experimental conditions. As illustrated in Figure S1, the inhibitory concentrations observed for MCF-7 (IC_50_ = 6.52 µM) and MDA-MB-231 (IC_50_ = 13.87 µM) were found to be similar. According to the principles of pharmacophore modeling, the antiproliferative effect may be attributable to antagonism of ERα. Amodiaquine has been demonstrated to exert a cytotoxic effect on a range of BC cell lines, including MCF-7, MDA-MB-231, BT-549, and SK-BR-3. In addition, it has been demonstrated to impede the formation of colonies and the migration of cells, induce apoptosis, impede cell cycle progression, inhibit the expression of cancer-related genes, and induce autophagy inhibition by the LC3BII protein [93,94].

## 3. Materials and Methods

The methodological approach involved building multiple pharmacophoric models of ERβ agonist ligands and ERα antagonist molecules based on crystal structures deposited in the Protein Data Bank (PDB). Subsequently, the individual models of each complex were aligned to identify shared pharmacophoric features and unify them into a common model. The multicomplex pharmacophoric model was validated by evaluating its ability to discriminate between ERβ agonists and ERα agonist/antagonists. This model subsequently played a crucial role in drug selection from the FDA database and DrugBank. Finally, molecular docking simulations were employed to assess the capacity of the selected drugs to interact with the active site of ERβ and ERα. This analysis focused on the key interactions that underpin the agonist and antagonist activity of these drugs on both BC therapeutic targets (Figure 1).

### 3.1. Structural Data Collection

A comprehensive collection of structural data about both ER ligands and crystalline ligand-receptor complexes was assembled. In the initial stages of the investigation, a database called “ERβ training ligands” (TLERβ) was built from the bibliographic search of selective ligands to ERβ, with biological activity reported in clinical trials, pre-clinicals, or in vitro evaluations. Furthermore, a database called “ERα training ligands” (TLERα) reported with greater selectivity to ERα was performed to contrast with the value of adjustment of TLERβ ligands in the model used (all training ligands used are described in the Table S3). The search was carried out in databases such as: DrugBank (https://go.drugbank.com/ accessed on 1 August 2023); ChEMBL (https://www.ebi.ac.uk/chembl/ accessed on 5 August 2023) and Binding Database (https://www.bindingdb.org/rwd/bind/index.jsp, accessed on 10 August 2023). Once the bioactive ligands for each receptor were selected, the 2D chemical structures were constructed with the ChemDraw Professional 15.0 program and then converted to a 3D format using the Chem3D tool. This was followed by a structural minimization with molecular mechanics algorithms (MM2), in addition to geometric pre-optimization with the Merck molecular force field (MMFF94), including a dielectric constant of ε = 80.

The set of molecules used in the virtual screening phase was obtained from drug databases with fully described pharmacological and toxicological information. For this purpose, information from the database of drugs approved by the FDA (https://www.fda.gov/drugs/drug-approvals-and-databases/ (accessed on 25 March 2022)) and DrugBank (https://go.drugbank.com/releases/latest (accessed on 17 February 2022)) was used. The FDA database contains more than 1859 drugs (until 2021), while DrugBank contains more than 9249 (until 2021). For each database, prior to virtual screening, it was necessary to incorporate the molecules into a data file with the extension “.ldb” by converting the 2D structure to the 3D structure through energy minimization using the MMFF94s force field. The conformational search was then performed for each molecule using the iCon conformation generator in LigandScout 4.4 Advance, configured with the best search quality (the maximum number of conformations was 300 and the RMS threshold was 0.8 Å, excluding duplicate conformations) [95]. Structural data of the ligand-receptor crystalline complexes were obtained from the protein data bank PDB (https://www.rcsb.org/ (accessed on 19 September 2023)).

### 3.2. Structure-Based Pharmacophore Modeling

Structure-based pharmacophore modeling of ERβ was performed using the structural data of eighteen crystalline complexes obtained from PDB (https://www.rcsb.org/, accessed on 19 September 2023), including: 1U3Q, 1U3R, 1U3S, 1U9E, 1X76, 1X7B, 1X7J, 1X78, 1YYE, 1ZAF, 2GIU, 2JJ3, 2NV7, 2QTU, 2YLY, 2Z4B, 3OLS, and 7XVY. While for ERα the following crystal complexes were used: 1A52, 2P15, 2QE4, 3DT3, 3UUC, 4DMA, 4MGB, 4TUZ, 4TV1, 4ZN7, 5DUE, 5DXR, 5DZ0, 5E14, 5KRA and 5KRC. The pharmacophoric model was generated for each selected crystal complex using the software LigandScout 4.4 Advanced; they were then identified and aligned the matching pharmacophoric features based on the functional group, geometric distribution, and type of interactions with amino acid residues at the binding site as reference points [96]. Given that certain ERβ agonists demonstrate affinity for the ERα receptor, the selectivity correlation ratio Fold = KDERα/KDERβ was calculated using relative affinity values obtained from Binding DB (bindingdb.org, accessed on September 25, 2023). The agonists with the highest affinity ratio to ERβ (1U3R, 1U9E, 1X76, 1X78, 1X7B, 1X7J, 1YYE, 2GIU, 2JJ3, 2QTU, 2YLY, and 7XVY) were selected as reference compounds to identify the coincident and determining interactions for the agonist effect, in the pharmacophoric modeling. Additionally, to add a theoretical selectivity filter, a multi-complex pharmacophore model targeting ERα was constructed to compare and validate the results obtained. The pharmacophore features of each crystal complex were merged into a multicomplex pharmacophore model for both ERβ (MPMERβ) and ERα (MPMERα). This process was performed using the automated “Align and Merge Pharmacophores” tool in LigandScout 4.4 Advanced. The process involves aligning all elements, merging all features into a single model, and interpolating any overlapping features [97].

#### Multicomplex Pharmacophore Model Validation

To test the predictive and discriminative capacity of the pharmacophoric models based on the features required according to the MPMERβ and MPMERα hypothesis, a validation process was performed by virtual screening. First, the mapping was performed by filtering the set of TLERβ and TLERα ligands on the pharmacophoric interaction map of ERβ agonists and subsequently on the MPMERα, using LigandScout 4.4 Advanced [98]. In this virtual screening cross-validation process, the pharmacophore fit scores obtained in each model were compared as a measure of the correlation between the reported pharmacological activity of the ligands and the molecular interactions defined by each model.

Quantitative validation of the multi-complex model (MPMERβ) was performed with ROC (Receiver Operating Characteristic) curve analysis generated in LigandScout 4.4 software. A screening was performed comparing the databases of active estrogen receptor modulators (agonists and antagonists) and decoys (inactive molecules) retrieved from the DEKOIS 2.0 database of the University of Tübingen [99]. The Pharmacophore-Fit scoring function of the best-fitting conformer was applied, considering exclusion volumes. The ROC curve graph was obtained, along with the area under the curve (AUC) value, and the positive predictive value of the model was calculated. Sensitivity, or the percentage of recovered assets, was plotted on the *y*-axis. The complement of specificity, or the percentage of recovered decoys or false positives, was plotted on the *x*-axis. The AUC indicator was used to measure the test’s ability to discriminate between active and inactive compounds on ERβ. Values between 0 and 0.5 indicate random discrimination; values between 0.51 and 0.7, acceptable discrimination; values between 0.71 and 0.8, good discrimination; and values between 0.81 and 1.0, excellent model performance [100].

### 3.3. Multicomplex Pharmacophore-Based Virtual Screening

The validated MPMERβ pharmacophoric model was used in virtual screening with LigandScout 4.4 Advanced, filtering DrugBank and FDA drug libraries. This process allowed the identification of drugs that exhibited structural complementarity with the geometric and spatial arrangement of molecular interactions defined by the MPMERβ model. The focus was on identifying drugs that shared minimal molecular features. Search parameters during virtual screening were considered in advanced functions. Drugs were prioritized according to the “pharmacophore-fit” scoring function in the “get best matching conformation” screening mode [98], stopping the search until a match was found with all the query characteristics (at least 7 out of 10) and penalizing those molecules that interfere with the limits established for the exclusion volumes “check exclusion volume” at the active site. Subsequently, to obtain a discriminatory probabilistic result from the ligands identified in the first virtual screening, a cross-virtual screening was simulated using both maps (MPMERβ and MPMERα), considering a more demanding screening mode called “Fragment Screening”.

### 3.4. Molecular Docking

Blind docking was performed on both ERs using the Autodock Vina 1.1.2 extension in LigandScout 4.4, which analyzes all possible ligand-protein interactions with a sufficiently large search box that encompasses the entire receptor. Affinity free energy (kcal/mol) is considered by the highest-scoring conformers at the binding site. For reference and validation, the PDB ID: 1X7B structure (crystallized with Prinaberel) was used for the ERβ receptor, while the PDB ID: 3DT3 structure (crystallized with GW368) was used for ERα. Non-structural crystallographic water molecules, ions, and undesired co-crystallized ligands were removed. Protonation states were adjusted to pH 7.4, and polar hydrogens were added. The conformational geometry of the ligands was minimized using a molecular mechanic’s force field (MM2), and geometric optimization was then performed using Merck’s molecular force field (MMFF94), including a dielectric constant of ε = 80.0. The structures were protonated at pH 7.4 and assigned free rotatable bonds and Gasteiger charges. The receptor was treated as rigid, while all ligand rotatable bonds were allowed to rotate freely. All protein–ligand docking calculations were carried out using the Lamarckian Genetic Algorithm (LGA). The docking protocol was run with a thoroughness of 100, a maximum of 20 output binding modes, and an energy range of 3.0 kcal/mol. The highest-ranking conformations were selected based on predicted exergonic energy and structural inspection of key interactions. After the docking simulations were completed, the conformation with the lowest binding energy and the most populated cluster was selected as a hit.

As part of the optimization and validation process of the molecular docking strategy, the next step was to perform a site-directed (focused) docking using the AutoDock4 software [101]. This process consisted of evaluating ligand-receptor interactions at the known co-crystallized binding site on both receptors. The receptor structure was imported into AutoDockTools in PDB format, and missing bonds were generated using the Build by Distance option. Polar hydrogens were added, non-polar hydrogens were merged, and Koltman charges were assigned. Atom types corresponding to the AutoDock4 force field were then set using Assign AD4 Type. The fully prepared receptor was subsequently saved in PDBQT format. The grid box was configured in the Grid Box panel by adjusting its center and dimensions (x, y, z). A restricted search box of 216,000 Å^3^ was defined for the focused docking experiment, with grid points spaced at 0.375 Å intervals. For ERβ included amino acids: Leu298, Glu305, Met336, Ile373, His475, with Grid Box coordinates of *x* = 30.446, *y* = 37.028, *z* = 39.151 and for the ERα, the amino acids: Leu346, Glu353, Leu384, Met421, His524, with Grid coordinates of *x* = 40.539, *y* = −3.425, and *z* = 15.922, the AutoDockTools 1.5.6 software (https://ccsb.scripps.edu/mgltools/1-5-6/ (accessed on 17 February 2024)) was used to set those parameters, as well as analysis of results. The search parameters configured from the genetic algorithm before docking were: 100 runs of the algorithm, with a population size of 100, with a maximum of 1 × 10^7^ evaluations, with a maximum of 27000 generations, with 100 generations to choose the worst individual, while the other parameters were left default by the software. Finally, all ligand and protein .pdbqt files, along with the corresponding .gpf and .dpf parameter files, were placed in the same directory. The docking workflow was executed using the Windows command terminal by running autogrid4 (autogrid4.exe -p name.gpf -l name.glg) followed by Autodock4 (autodock4.exe -p name.dpf -l name.dlg). Upon completion, a .dlg output file was generated, containing all data related to the docking analysis. The energetic results and molecular interactions generated by the reference drugs and the selected drugs were analyzed using MGLTools 1.5.6, Chimera 1.17 (https://www.rbvi.ucsf.edu/chimera/ (accessed on 17 February 2024)), and BIOVIA Discovery Studio 21.1 (https://discover.3ds.com/discovery-studio-visualizer-download (accessed on 17 February 2024), academic license).

To validate the targeted molecular docking parameters, self-docking was performed using the co-crystallized ligand for each receptor: Prinaberel (ERβ agonist) and GW368 (ERα agonist) (see Table S7). The resulting conformations were then compared with the bioactive conformation using root mean square deviation (RMSD) to assess docking accuracy with an RMSD tolerance of less than 2 Å [102]. The RMSD calculation to validate the binding by orientation of the ligand at the active site was performed using DockRMSD (https://aideepmed.com/DockRMSD/ accessed on 10 August 2024).

In the final weighting of the virtual screening, the calculation of the theoretical ERβ selectivity index (TSI ERβ) was performed by summing the ERβ/ERα ratio obtained by the pharmacophore fit score (PFSc ERβ/ERα_drug_) and by the affinity energy (AE ERβ/ERα_drug_), minus the result of this summation obtained by Estradiol (PFS ERβ/ERα_estradiol_ + AE ERβ/ERα_estradiol_), considering the following expression:

(PFSc ERβ/ERα_drug_ + AE ERβ/ERα_drug_) − (PFSc ERβ/ERα_estradiol_ + AE ERβ/ERα_estradiol_).

Values > 0 represent theoretical selectivity for ERβ, which was observed with the selective agonists. Values < 0 were obtained with ligands that showed experimental affinity for ERα.

### 3.5. Cell Culture

The BC cell lines used in this study were MCF-7 and MDA-MB-231, obtained from the American Type Tissue Culture Collection (ATCC, Rockville, MD, USA). The cells were routinely cultured in 100 mm × 20 mm Petri dishes, with Dulbecco’s modified Eagle Medium (DMEM) high glucose (4.5 g/L), without phenol red (Gibco, Waltham, MA, USA). The medium for the MCF-7 cell line was supplemented with 7% bovine fetal serum (Gibco, Waltham, MA, USA) and the medium for MDA-MB-231 was supplemented with 10% SFB, in both 1% antibiotic was added (Penicillin/Streptomycin (10,000 U/mL, Gibco, Waltham, MA, USA). and 1% Non-essential Amino Acids (Gibco, Waltham, MA, USA). Cell cultures were incubated at 37 °C in a humidified atmosphere of 5% CO_2_ and 95% air.

### 3.6. Cell Viability Assay

In the MTT [3-(4,5-dimethylthiazol-2-yl)-2,5-diphenyl-tetrazolium bromide] (Sigma-Aldrich, Burlington, MA, USA) metabolic cell viability assay, MTT is reduced by the activity of mitochondrial succinate dehydrogenase, to produce formazan (purple), which makes it possible to determine mitochondrial functionality. The amount of formazan formed is proportional to the number of living cells present in each well [103]. For the assay, an MTT stock solution (5 mg/mL) was freshly prepared in phosphate-buffered saline (PBS) and stored in the dark until use. Cells were grown until approximately 70% confluence, then detached using 2 mL of trypsin (1%)–EDTA (0.53 mM) (Gibco, Waltham, MA, USA) for 4 min at 37 °C. The cell suspension was collected in 2 mL of complete medium, centrifuged at 3 × 10^3^ rpm for 10 min, and resuspended in 2 mL of fresh medium. Cell numbers were determined using an automated cell counter (Corning^®^ Cell Counter, Somerville, NY, USA). For viability assays, MDA-MB-231 cells were seeded at a density of 9.5 × 10^3^ cells per well and MCF-7 cells at a density of 7.5 × 10^3^ cells per well in 96-well plates. (NEST Biotechnology Co., Wuxi, China). After 24 h of incubation at 37 °C with 5% CO_2_ to allow cell adherence, the cells were treated with increasing concentrations of the reference compound (*S*-DPN) and the candidate drugs for 48 h. After the 48 h drug exposure period, 20 µL of the MTT solution was added to each well of the 96-well plate, followed by incubation for 2.5 h at 37 °C in a humidified atmosphere of 5% CO_2_. During this period, the yellow MTT salt was taken up by the cells and enzymatically reduced to formazan. After incubation, the MTT-containing medium was carefully aspirated to avoid disturbing the formed crystals. The formazan precipitate was then solubilized by adding 100 µL of dimethyl sulfoxide (DMSO, Sigma-Aldrich, Burlington, MA, USA) to each well. Plates were gently shaken for 5–10 min to ensure complete dissolution of the crystals and to achieve a homogeneous color distribution. Absorbance was measured at 550 nm using a microplate reader (MultiSkan, Thermo Scientific, Waltham, MA, USA). Cell viability (%) was calculated relative to vehicle-treated controls (culture medium containing 0.5% DMSO), which were defined as 100% viability. All conditions were evaluated with six technical replicates in three independent biological experiments. The half-maximal inhibitory concentration (IC_50_) and standard error (SE) were determined using nonlinear regression analysis with a sigmoidal dose–response model (four parameter logistic curve) incorporating a Hill slope. All curve-fitting procedures were performed using GraphPad Prism version 8.4.3 (GraphPad Software, San Diego, CA, USA).

## 4. Conclusions

The process of drug discovery for BC is subject to constant evolution, and the utilization of in silico tools holds the potential to accelerate it. In this regard, the identification of pharmacophoric features based on crystal complexes of estrogen receptors (ERα and ERβ) co-crystallized with selective ligands has allowed for the identification of drugs that meet the structural and molecular recognition requirements, topologically established by ERβ agonists, through virtual screening based on multi-complex pharmacophoric modeling. This chemoinformatics strategy not only facilitates the exploration of the possible repurposing of drugs as anticancer agents but also provides valuable information for the rational de novo design of compounds inspired by the pharmacophore requirements structure based off ERs. Using this approach, we identified Sobetirome, Labetalol, and Procaterol as promising antiproliferative agents on BC cell lines. Procaterol demonstrated the most significant antiproliferative activity in vitro, with IC_50_ values of 21.26 and 36.10 µM in the MCF-7 and MDA-MB-231 cell lines, respectively. Overall, these results are encouraging, but they require experimental validation and additional preclinical studies to clarify the mechanisms associated with ERβ activation and define an effective and safe dose that supports its possible repositioning as a therapeutic alternative for BC.

## Figures and Tables

**Table 1:** Top-ranked drugs in virtual screening based on pharmacophoric modeling and molecular docking.

Drug	Pharmacophore Fit Score (PFSc) *^a^*			Affinity Energy *^b^*			TSI *^c^*	Therapeutic Target *^d^*
	ERβ	ERα	ERβ/ERα	ERβ	ERα	ERβ/ERα		
CHEMBL193064	106.94	66.73	1.60	−10.38	−9.16	1.13	0.63	ERβ agonist (ERβ Ref)
DB06875	98.03	67.77	1.45	−10.29	−9.15	1.12	0.46	ERβ agonist
S-DPN	87.98	80.06	1.10	−8.77	−8.24	1.06	0.05	ERβ agonist (ERβ Ref)
DB06832	86.75	66.55	1.30	−9.03	−8.27	1.09	0.29	ERβ agonist
DB07425	85.30	76.38	1.12	−9.70	−8.44	1.15	0.16	TRβ1 agonist
DB14008	77.82	77.12	1.01	−8.59	−8.62	1.00	−0.10	JAK2/STAT3 inhibitor
DB01645	77.33	57.51	1.34	−9.32	−8.35	1.12	0.35	ERβ agonist
Raloxifene	76.87	96.82	0.79	−8.86	−13.66	0.65	−0.67	ERα antagonist
Bazedoxifene	76.83	106.45	0.72	−8.88	−13.40	0.66	−0.73	ERα antagonist
Diflunisal	76.82	76.76	1.00	−6.74	−6.38	1.06	−0.05	PTGS 1/2 inhibitor
Labetalol	76.64	58.62	1.31	−9.51	−8.85	1.07	0.27	Mixed β and αAR antagonist
Procaterol	76.38	75.52	1.01	−8.71	−7.61	1.14	0.05	β2AR agonist
GW368	75.60	105.85	0.71	−9.91	−10.80	0.92	−0.48	ERα agonist (ERα Ref)
DB00179	75.58	87.33	0.87	−9.56	−9.35	1.02	−0.22	5-LO inhibitor
Dobutamine	75.55	76.70	0.99	−8.87	−8.46	1.05	−0.08	β1AR agonist
DB15058	67.88	76.58	0.89	−8.67	−7.72	1.12	−0.10	Selective affinity for βA-plaques
Fenoldopam	67.56	96.39	0.70	−8.14	−7.67	1.06	−0.35	D1 agonist
Ritodrine	67.07	85.62	0.78	−8.42	−8.37	1.01	−0.32	β2AR agonist
DB14129	66.50	76.42	0.87	−10.40	−9.20	1.13	−0.11	NSAID and neuroprotective
DBMET00290	57.36	67.13	0.85	−10.93	−9.80	1.12	−0.14	Heme polymerase inhibitor
Estradiol	56.89	57.09	1.00	−10.74	−9.67	1.11	0.00	Endogenous estrogen
Capsaicin	55.75	66.91	0.83	−8.96	−8.11	1.10	−0.17	TRPV1 agonist
Arbutamine	54.86	57.43	0.96	−8.46	−7.94	1.07	−0.09	β1AR agonist
Formoterol	54.68	57.94	0.94	−8.88	−8.27	1.07	−0.09	β2AR agonist
Nadolol	46.57	66.10	0.70	−9.15	−8.15	1.12	−0.28	β2 and β1AR antagonist
Fulvestrant	46.54	45.02	1.03	−10.91	−12.10	0.90	−0.17	ERα degrader
Amodiaquine	37.36	67.42	0.55	−9.92	−9.26	1.07	−0.48	PfPMT inhibitor
DB04468	>35.0	86.69	0.40	−9.49	−10.70	0.89	−0.82	ERα antagonist
PPT	>35.0	87.22	0.40	−10.06	−10.50	0.96	−0.75	ERα agonist (ERα Ref)
Pravastatin	>35.0	76.45	0.46	−10.39	−9.02	1.15	−0.50	HMG-CoA red inhibitor
Dipivefrin	>35.0	55.97	0.63	−9.93	−9.34	1.06	−0.42	β2 and αAR agonist

*^a^* Match the fit value to the pharmacophoric characteristics obtained by screening in LigandScout. *^b^* Free energy obtained by guided docking in AutoDock4. *^c^* Theoretical Selectivity Index for ERβ: (PFSc ERβ/ERα_drug_ + AE ERβ/ERα_drug_) − (PFSc ERβ/ERα_estradiol_ + AE ERβ/ERα_estradiol_). *^d^* Therapeutic information obtained from DrugBank.org.

**Table 2:** Antiproliferative activity on BC cells of the drugs selected in the virtual screening.

Antiproliferative Activity IC_50_ (µM)		
Drug	MCF-7	MDA-MD-231
*S*-DPN	122.50 ± 3.09	186.85 ± 3.83
Sobetirome	170.50 ± 4.13	206.00 ± 2.59
Labetalol	127.50 ± 2.35	146.30 ± 3.10
Procaterol	21.26 ± 3.27	36.10 ± 2.92

## Data Availability

The original contributions presented in this study are included in the article/supplementary material. Further inquiries can be directed to the corresponding authors.

## Supplementary Materials

The following supporting information can be downloaded at: https://www.mdpi.com/article/10.3390/ijms27010463/s1. Refs. [45,104,105,106,107,108,109,110,111,112,113,114,115,116,117,118,119,120,121,122,123,124] are cited in Supplementary Materials file.

## Abbreviations

The following abbreviations are used in this manuscript:

AMPK	AMP-activated protein kinase
AUC	Area under the curve
E2	17β-Estradiol
EGFR	Epidermal growth factor receptor
ERs	Estrogen receptors
ERα	Estrogen receptor alpha
ERβ	Estrogen receptor beta
FBS	Fetal bovine serum
HBA	Hydrogen bond acceptor
HBD	Hydrogen bond donor
LBD	Ligand-binding domain
MPMERα	Multicomplex pharmacophore model for ERα
MPMERβ	Multicomplex pharmacophore model for ERβ
MTT	3-(4,5-Dimethylthiazol-2-yl)-2,5-diphenyl tetrazolium bromide
PDB	Protein data bank
PfPMT	Phosphoethanolamine methyltransferase
PPT	Propyl pyrazole triol
ROC	Receiver operating characteristic
TSI	Theoretical selectivity index
ΔG	Binding free energy change (kcal/mol) in molecular docking
