# Supplementary material for: Multicomplex Pharmacophore Modeling of Estrogen Receptors Suggests the Probable Repurposing of Procaterol as an Antiproliferative Agent Against Breast Cancer Cells
Source: Int J Mol Sci. 2026 Jan 1;27(1):463. doi: 10.3390/ijms27010463 (PMC12786801; doi:10.3390/ijms27010463)
Supplement: Supplementary file 1 [file ijms-27-00463-s001.zip › ijms-4066923-supplementary.pdf]

International Journal of *Molecular Sciences*

MDPI

Supplementary Materials

Multicomplex Pharmacophore Modeling of Estrogen Receptors Suggests the Probable Repurposing of Procaterol as an Anti-proliferative Agent Against Breast Cancer Cells

Luis Heriberto Vazquez-Mendoza[1], Humberto L. Mendoza-Figueroa[1]*, Nadia Judith Jacobo-Herrera[2], Norbert Bakalara[3], Daphne Edith González-Juárez[4], José Correa-Basurto[1] and Juan Benjamín García-Vázquez[1,5]*

[1] Laboratorio de Diseño y Desarrollo de Nuevos Fármacos e Innovación Biotecnológica (Laboratory for the Design and Development of New Drugs and Biotechnological Innovation), Escuela Superior de Medicina del Instituto Politécnico Nacional (ESM-IPN), Plan de San Luis y Salvador Díaz Mirón S/N, Casco de Santo Tomás, CDMX, 11340, México; lvazquezm1903@alumno.ipn.mx (L.H.V.M.); jcorreab@ipn.mx (J.C.B.).

[2] Unidad de Bioquímica, Instituto Nacional de Ciencias Médicas y Nutrición Salvador Zubirán, CDMX, 14080, México; nadia.jacoboh@incmnsz.mx (N.J.J.H.).

[3] Ecole Nationale Supérieure de Technologie des Biomolécules de Bordeaux (ENSTBB), Université de Bordeaux, CNRS, Bordeaux INP, CBMN, UMR 5248, F-33600 Pessac, France; norbert.bakalara@enstbb.fr (N.B.).

[4] División de Biología Molecular, Instituto Potosino de Investigación Científica y Tecnológica (DBM-IPICYT), y Secretaría de Ciencia, Humanidades, Tecnología e Innovación (SECIHTI), San Luis Potosí 78216, SLP, México; daphne.gonzalez@ipicyt.edu.mx (D.E.G.J.).

[5] Investigadoras e Investigadores por México SECIHTI -Sección de Estudios de Posgrado e Investigación de la Escuela Superior de Medicina, Instituto Politécnico Nacional, Plan de San Luis y Salvador Díaz Mirón S/N, Casco de Santo Tomás, CDMX, 11340, México.

Contents:

- **Table S1.** Individual structure-based pharmacophore modeling of the eighteen crystals ERβ analyzed.
- **Table S2.** Individual structure-based pharmacophore modeling of the sixteen crystals of ERα analyzed.
- **Table S3.** Training estrogenic ligands for validating multicomplex pharmacophore models.
- **Table S4.** Pharmacophore fit score of training ligands (TLERβ and TLERα) in the cross-validation of the MPMERβ and MPMERα multicomplex pharmacophore models.
- **Table S5.** Top-ranked compounds in the validation screening of the active molecules and decoys databases, employing the DEKOIS 2.0 database
- **Table S6.** Structural conformer with the best MPMERβ pharmacophore fit score, identified in the virtual screening of the FDA and DrugBank databases.
- **Table S7**. RMSD values of the conformational overlap of the co-crystallized ligand (blue) and the theoretical conformer (olive green) obtained by molecular docking.
- **Table S8.** Interaction energy and 2D diagram of molecular interactions of drugs selected by virtual screening on MPMERβ, using molecular docking.
- **Figure S1.** Antiproliferative activity of Amodiaquine on the MCF-7 and MDA-MB-231 cell lines.
- **References**

Table S1. Individual structure-based pharmacophore modeling of the eighteen crystals ERβ analyzed. The binding affinity and relative affinity values of the co-crystallized agonists are shown.

PDB ID	Agonist (co-crystallized)	SB-Pharmacophore model	Binding affinity (EC_{50})	Selectivity rate $(ER\beta/ER\alpha)^{-1}$	References
**3OLS**	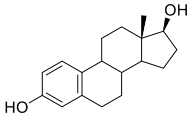 **Estradiol**	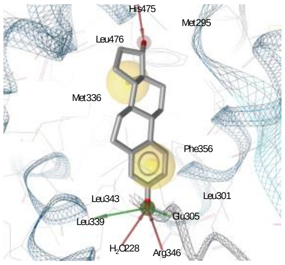	β= 2 nM<br>α= 2 nM<br><br>β= 1.35 nM<br>α= 1.20 nM	~1	[104,105]
**1U3S**	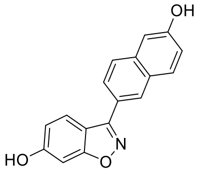 **WAY-797**	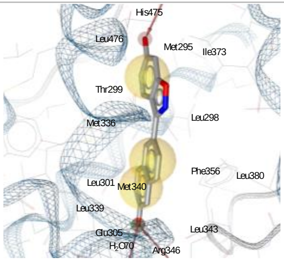	β= 1.4 nM<br>α= 8 nM	5.71	[106]
**1U3Q**	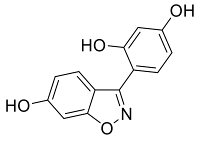 **CL-272**	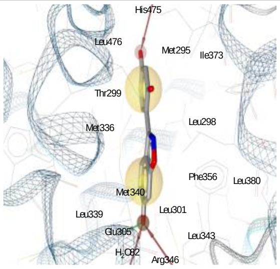	β= 3.5 nM<br>α= 24 nM	6.86	[106]
**1ZAF**	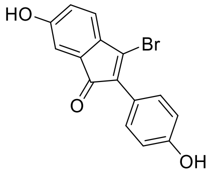 **789**	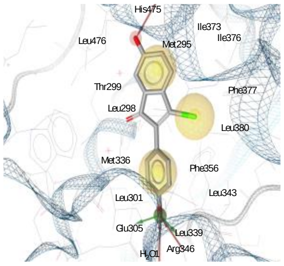	β= 20 nM<br>α= 290 nM	14.5	[107]
**2NV7**	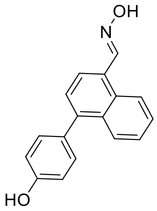 **WAY555**	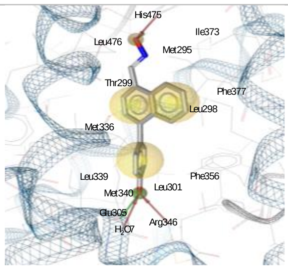	β= 5 nM<br>α= 95 nM	19	[108]

International Journal of
Molecular Sciences

MDPI

**2Z4B**	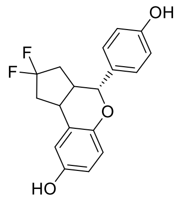 **DC8**	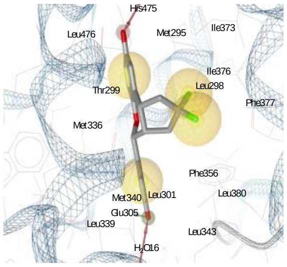	β= 0.44 nM<br>α= 8.4 nM	19.09	[109]
**1X7J**	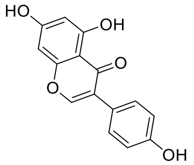 **Genistein**	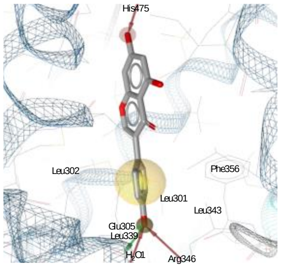	β= 200 nM<br>α= 3920 nM	19.6	[110]
**1X76**	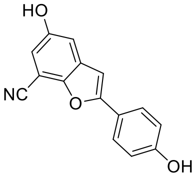 **WAY-697**	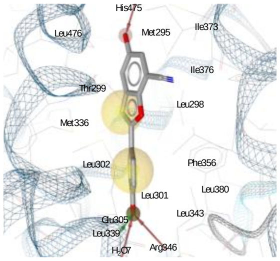	β= 2.2 nM<br>α= 46 nM	20.91	[111]
**1U3R**	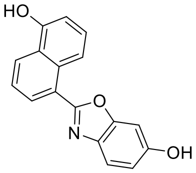 **WAY388**	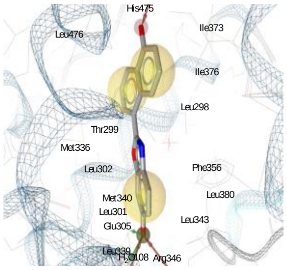	β= 5 nM<br>α= 117 nM	23.4	[106]
**1U9E**	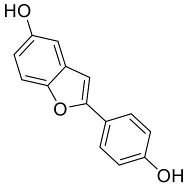 **WAY-397**	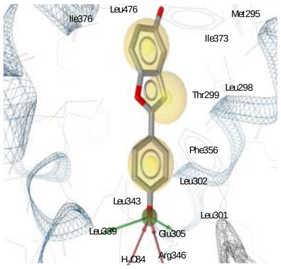	β= 6 nM<br>α= 176 nM	29.33	[111]
**2JJ3**	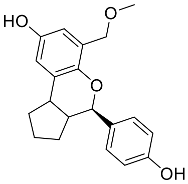 **JJ3**	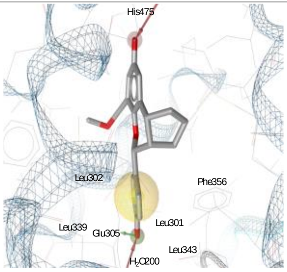	β= 0.28 nM<br>α= 11.9 nM	42.5	[109]

International Journal of *Molecular Sciences*

MDPI

**2YLY**	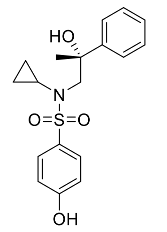 **SU4**	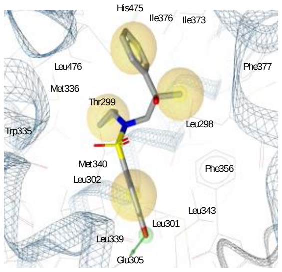	β= 79 nM<br>α= 5400 nM	68.35	[112]
**7XVY**	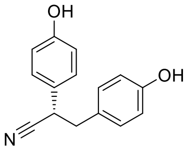 **S-DPN**	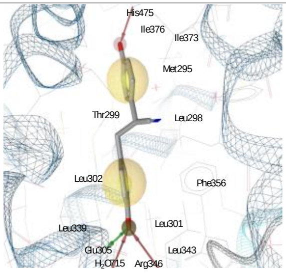	β= 66 nM<br>α= 0.85 nM	77.64	[113]
**1YYE**	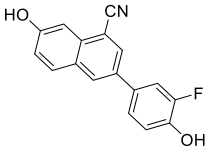 **WAY202196**	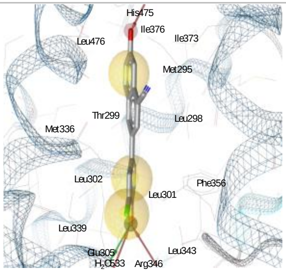	β= 2.7 nM<br>α= 210 nM	77.78	[114]
**1X78**	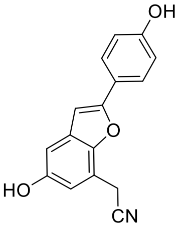 **WAY244**	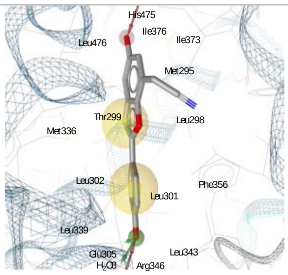	β= 14 nM<br>α= 1150 nM	82.14	[111]
**2QTU**	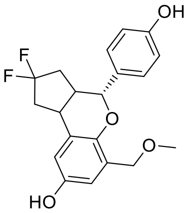 **3AS**	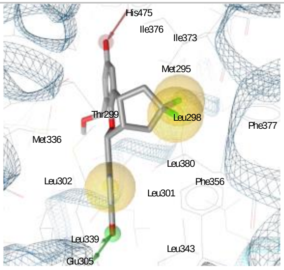	β= 0.53 nM<br>α= 43.8 nM	82.64	[109]
**2GIU**	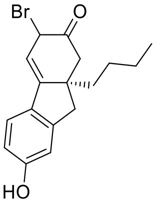 **FBR**	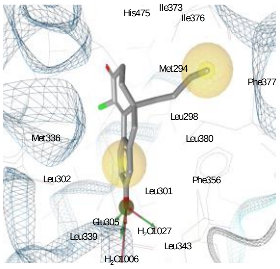	β= 1.5 nM<br>α= 86 nM	86.00	[115]

International Journal of
Molecular Sciences

MDPI

**1X7B**	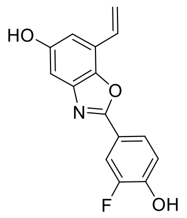 **Prinaberel**	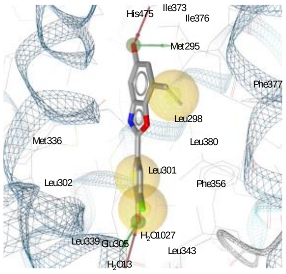	β= 3.1 nM<br>α= 618 nM	199.35	[106]

Data collected from "Protein Data Bank" and "The Binding Database". The pharmacophore model was created using Ligandscout software.

Table S2. Individual structure-based pharmacophore modeling of the sixteen crystals of ER**α** analyzed. The binding affinity and relative affinity values of co-crystallized ligands are shown.

PDB ID	Ligand (co-crystallized)	SB-Pharmacophore model	Binding affinity (EC_{50})	Selectivity rate $(ER\beta/ER\alpha)^{-1}$	References
**1A52**	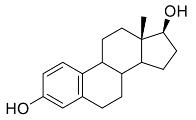 **Estradiol**	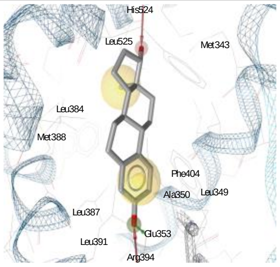	β= 2 nM<br>α= 2 nM<br><br>β= 1.35 nM<br>α= 1.20 nM	~1	[116]
**2P15**	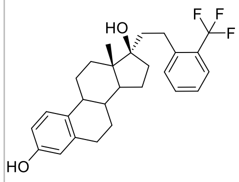 **EZT**	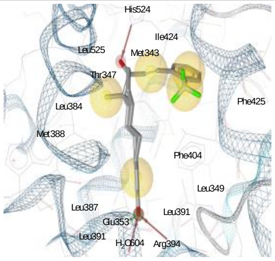	α= 50 pM<br>β= --	Not reported	[117]
**2QE4**	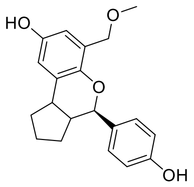 **JJ3**	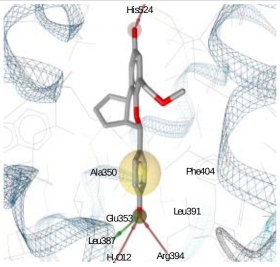	α= 0.28 nM<br>β= 11.0 nM	39.29	[118]
**3DT3**	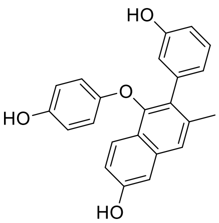 **GW368**	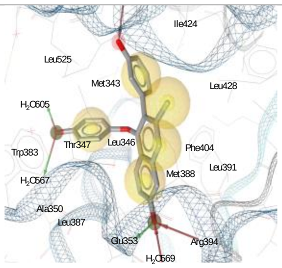	α= --<br>β= --	Not reported	[119]
**3UUC**	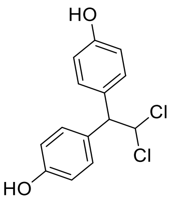 **0D1**	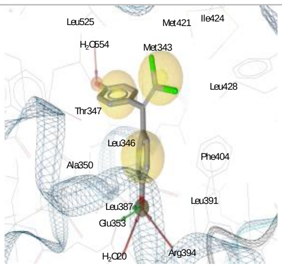	α= 1.9 nM<br>β= 3.4 nM	1.79	[120]

International Journal of
Molecular Sciences

MDPI

**4DMA**	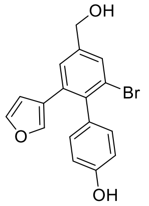<br>**0L8**	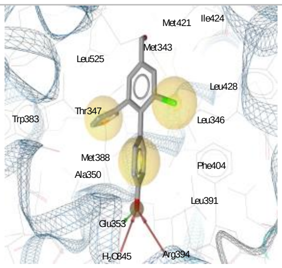	α= --<br>β= --	Not reported	[121]
**4MGB**	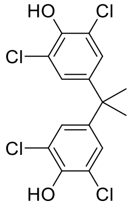<br>**TCBPA**	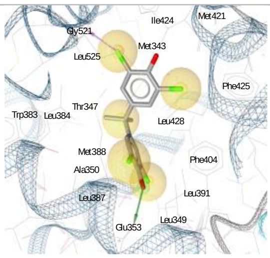	α= 11.7 μM<br>β= 68.0 μM	5.8	[122]
**4TUZ**	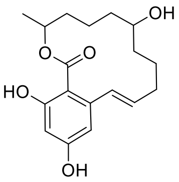<br>**36J**	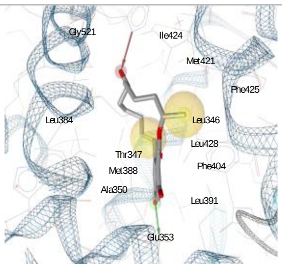	α= 0.01–1 nM<br>β= --	Not reported	[123]
**4TV1**	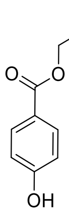<br>**36M**	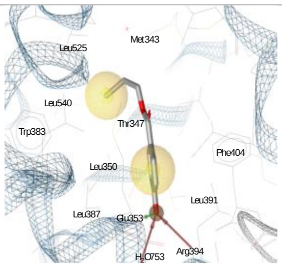	α= 3.55 μM<br>β= --	Not reported	[123]
**4ZN7**	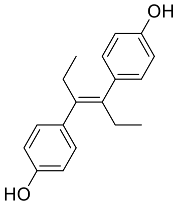<br>**Diethylstilbestrol**	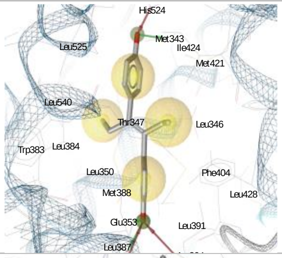	α= 0.49 nM<br>β= 0.63 nM	1.29	[46]
**5DUE**	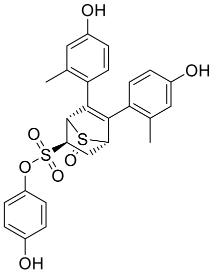<br>**5FY**	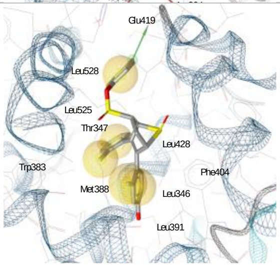	α= --<br>β= --	Not reported	[46]

**5DXR**	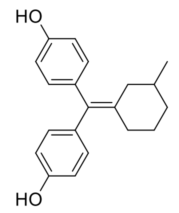 **5HW**	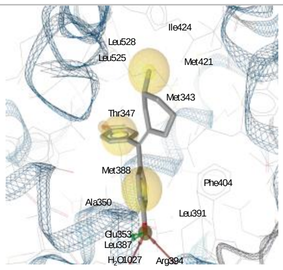	α= 124 nM<br>β= 285 nM	2.3	[46]
**5DZ0**	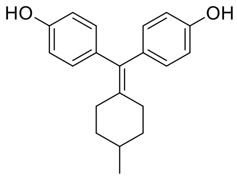 **5K0**	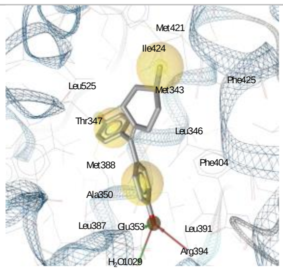	α= 66.5 nM<br>β= 274 nM	4.12	[46]
**5E14**	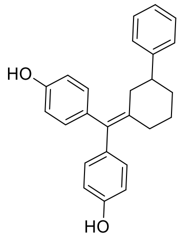 **5KB**	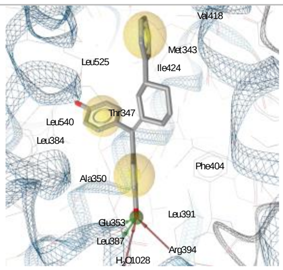	α= --<br>β= --	Not reported	[46]
**5KRA**	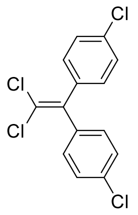 **6WS**	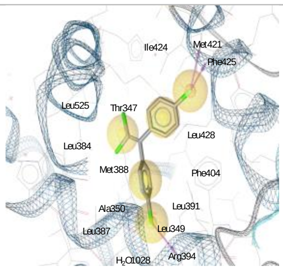	α= 4.36 μM<br>β= 69.07 μM	15.84	[124]
**5KRC**	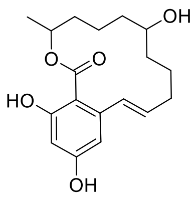 **Zearalenone**	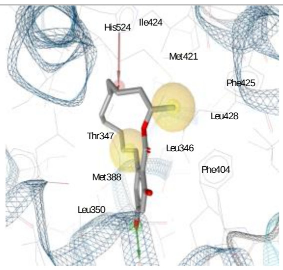	α= 240.4 nM<br>β= 165.7 nM	1.45	[124]

Data collected from "Protein Data Bank" and "The Binding Database". The pharmacophore model was created using Ligandscout software.

International Journal of *Molecular Sciences*

MDPI

Table S3. Training estrogenic ligands for validating multicomplex pharmacophore models. The blue box shows the ERβ training ligands (TLERβ), and the orange shaded box shows the ERα training ligands (TLERα).

**ERβ training ligands (TLERβ)**				
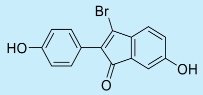<br>**1ZAF**	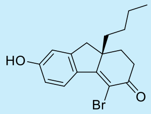<br>**2GIU**	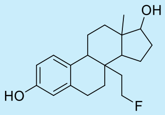<br>**8β-FEE2**	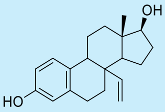<br>**8β-VE2**	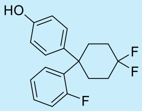<br>**AC-186**
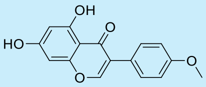<br>**Biochanine A**	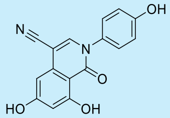<br>**β-LNGD1**	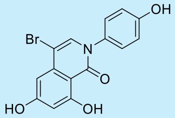<br>**β-LNGD2**	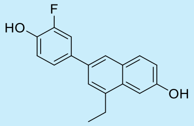<br>**CHEMBL193064**	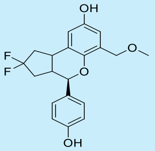<br>**CHEMBL236720**
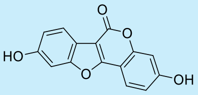<br>**Coumestrol**	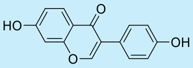<br>**Daidezein**	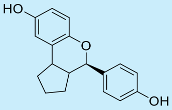<br>**Erteberel**	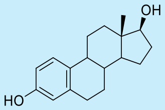<br>**Estradiol**	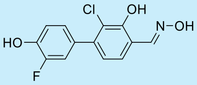<br>**FERb033**
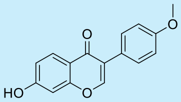<br>**Formononetin**	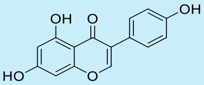<br>**Genistein**	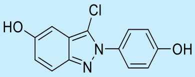<br>**Indazole-Cl**	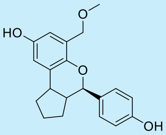<br>**JJ3**	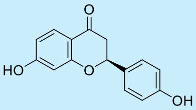<br>**Liquiritigenin**
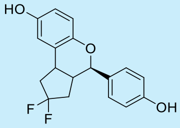<br>**LY3201**	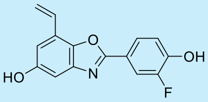<br>**Prinaberel**	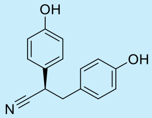<br>**R-DPN**	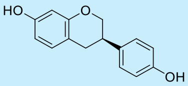<br>**R-Equol**	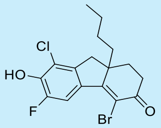<br>**SCHEMBL2417751**
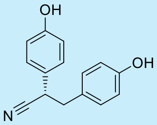<br>**S-DPN**	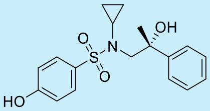<br>**SU4**	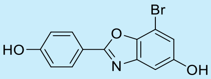<br>**WAY200070**	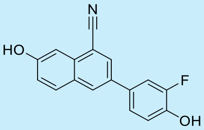<br>**WAY202196**	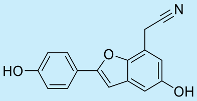<br>**WAY244**
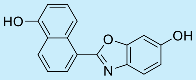<br>**WAY388**	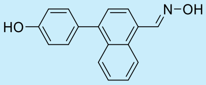<br>**WAY555**			

Table S3. *…continuation*

ERα training ligands (TLERα)				
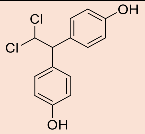<br>**0D1**	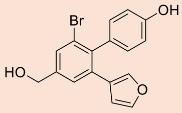<br>**0L8**	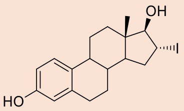<br>**16α-Iodo-E2**	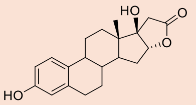<br>**16α-LE2**	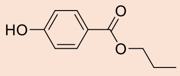<br>**36M**
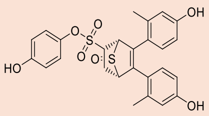<br>**5FY**	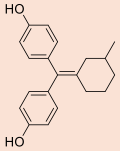<br>**5HW**	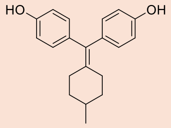<br>**5K0**	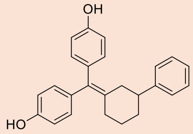<br>**5KB**	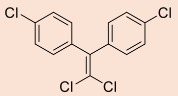<br>**6WS**
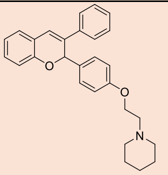<br>**CDRI-85/287**	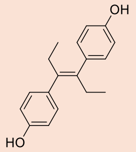<br>**Diethylstilbestrol**	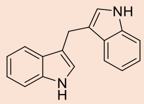<br>**DIM**	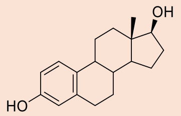<br>**Estradiol**	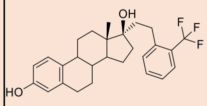<br>**EZT**
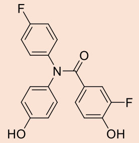<br>**GTx-758**	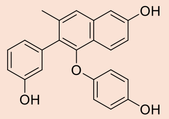<br>**GW368**	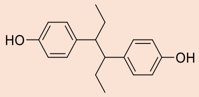<br>**Hexestrol**	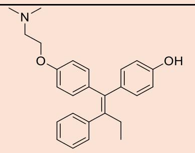<br>**Hydroxytamoxifen**	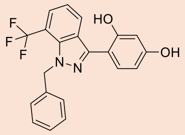<br>**KN0**
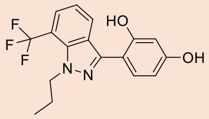<br>**KN1**	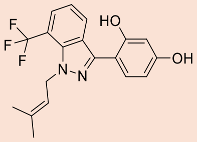<br>**KN3**	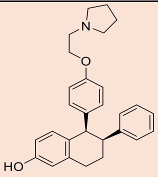<br>**Lasofoxifene**	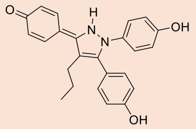<br>**Propylpyrazoletriol**	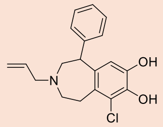<br>**SKF-82958**
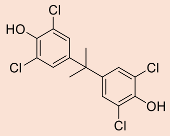<br>**TCBPA**	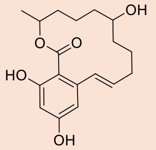<br>**Zearalenol**			

International Journal of Molecular Sciences MDPI

Table S4. Pharmacophore fit score of training ligands (TLERβ and TLERα) in the cross-validation of the MPMERβ and MPMERα multicomplex pharmacophore models. The MPMERβ and MPMERα models demonstrated the highest pharmacophore fit scores for those ligands that exhibited a correlation with their reported pharmacological activity.

	Drug	Affinity reported to ER	Pharmacophore-fit Score		Meet	Relative Pharmacophore-fit Score		Meet
			MPMERβ	MPMERα		MPMERβ	MPMERα	
1.	CHEMBL193064	β	106.94	66.73	✓	0.97	0.56	✓
2.	WAY202196	β	98.03	66.77	✓	0.89	0.56	✓
3.	LY3201	β	97.08	87.43	✓	0.88	0.73	✓
4.	Indazole-Cl	β	96.28	86.18	✓	0.88	0.72	✓
5.	FERb033	β	88.35	76.49	✓	0.80	0.64	✓
6.	Coumestrol	β	88.35	68.00	✓	0.80	0.57	✓
7.	B-LNGD2	β	88.23	65.86	✓	0.80	0.55	✓
8.	Daidezein	β	88.05	66.91	✓	0.80	0.56	✓
9.	Erteberel	β	87.82	65.88	✓	0.80	0.55	✓
10.	R-Equol	β	87.71	78.29	✓	0.80	0.65	✓
11.	S-DPN	β	87.63	86.06	✓	0.80	0.72	✓
12.	R-DPN	β	86.87	86.15	✓	0.79	0.72	✓
13.	Prinaberel	β	86.75	58.47	✓	0.79	0.49	✓
14.	CHEMBL236720	β	86.54	77.33	✓	0.79	0.64	✓
15.	WAY200070	β	86.31	66.72	✓	0.78	0.56	✓
16.	AC-186	β	86.20	85.41	✓	0.78	0.71	✓
17.	WAY388	β	86.03	77.01	✓	0.78	0.64	✓
18.	1zaf	β	85.81	76.43	✓	0.78	0.64	✓
19.	B-LNGD1	β	78.19	57.03	✓	0.71	0.48	✓
20.	Genistein	β	77.33	57.51	✓	0.70	0.48	✓
21.	Formononetin	β	67.71	65.39	✓	0.62	0.54	✓
22.	SCHEMBL2417751	β	66.86	55.70	✓	0.61	0.46	✓
23.	SU4	β	66.50	76.58	X	0.60	0.64	X
24.	WAY244	β	66.34	67.40	X	0.60	0.56	✓
25.	Liquiritigenin	β	66.08	77.04	X	0.60	0.64	X
26.	8β-VE2	β	65.86	67.55	X	0.60	0.56	✓
27.	Estradiol	β	56.89	57.09	✓	0.52	0.48	✓
28.	WAY555	β	56.45	68.08	X	0.51	0.57	X
29.	Biochanine A	β	46.22	48.97	X	0.42	0.41	✓
30.	8β-FEE2	β	--	77.52	X	--	0.65	X
31.	2giu	β	--	56.64	X	--	0.47	X
32.	JJ3	β	--	48.66	X	--	0.41	X
1.	GW368	α	75.60	105.85	✓	0.69	0.88	✓
2.	Hexestrol	α	87.82	105.83	✓	0.80	0.88	✓
3.	5FY	α	65.83	97.64	✓	0.60	0.81	✓
4.	Diethylstilbestrol	α	87.80	96.04	✓	0.80	0.80	✓
5.	GTx-758	α	87.24	95.83	✓	0.79	0.80	✓
6.	5HW	α	66.63	87.76	✓	0.61	0.73	✓

International Journal of *Molecular Sciences*

MDPI

7.	Propylpyrazoletriol	α	--	87.22	✓	--	0.73	✓
8.	5K0	α	57.17	87.09	✓	0.52	0.73	✓
9.	*Hydroxytamoxifen	α	--	86.69	✓	--	0.72	✓
10.	0D1	α	75.35	86.05	✓	0.68	0.72	✓
11.	**KN1	α	87.18	85.96	X	0.79	0.72	X
12.	**KN3	α	87.22	85.54	X	0.79	0.71	X
13.	KN0	α	77.26	85.39	✓	0.70	0.71	✓
14.	5KB	α	--	78.35	✓	--	0.65	✓
15.	*Lasofoxifene	α	--	77.88	✓	--	0.65	✓
16.	SKF-82958	α	77.31	76.80	X	0.70	0.64	X
17.	0L8	α	65.28	76.80	✓	0.59	0.64	✓
18.	Zearalenol	α	55.74	75.68	✓	0.51	0.63	✓
19.	XDH	α	56.54	75.35	✓	0.51	0.63	✓
20.	EZT	α	--	75.04	✓	--	0.63	✓
21.	36M	α	68.04	66.61	X	0.62	0.56	X
22.	Estradiol	α	56.89	57.09	✓	0.52	0.48	✓
23.	16α-LE2	α	35.58	56.57	✓	0.32	0.47	✓
24.	***CDRI-85/287	α	--	56.50	✓	--	0.47	✓
25.	16α-Iodo-E2	α	75.81	46.43	X	0.69	0.39	X
26.	6WS	α	--	46.23	✓	--	0.39	✓
27.	DIM	α	--	36.40	✓	--	0.30	✓

--Very low Score (not reported by Ligandscout), *ERα antagonist, **ERα partial agonist ***SERD

International Journal of
Molecular Sciences

Table S5. Top-ranked compounds in the validation screening of the active molecules and decoys databases, employing the DEKOIS 2.0 database.
Matching pharmacophore features **MPMERβ**: HBD: ■, HBA: ■, H: ■

	Compound	Matching features	Score	Active/Decoy
**1**	BDB50152609		98.18	active
**2**	BDB50168367		97.72	active
**3**	BDB50201159		97.43	active
**4**	BDB50310370		97.38	active
**5**	BDB50168331		97.24	active
**6**	BDB50161713		97.20	active
**7**	BDB50174924		97.20	active
**8**	BDB501166755		97.12	active
**9**	BDB50173663		96.54	active
**10**	BDB50123205		96.37	active
**11**	BDB50154137		96.33	active
**12**	BDB50104325		96.16	active
**13**	BDB50152616		96.00	active
**14**	ZINC28539264		95.50	*decoy*
**15**	BDB50154059		95.37	active
**16**	ZINC24798806		95.12	*decoy*
**17**	BDB50154134		88.42	active
**18**	BDB50154066		88.11	active
**19**	BDB19970		87.97	active
**20**	BDB19968		87.88	active
**21**	BDB50136241		87.40	active
**22**	BDB50157493		87.36	active
**23**	BDB50146281		87.03	active
**24**	BDB50180469		86.93	active
**25**	BDB17292		86.91	active
**26**	BDB50104326		86.53	active
**27**	BDB50104329		86.40	active
**28**	BDB50126769		86.27	active
**29**	BDB50264124		86.05	active
**30**	BDB50180478		77.94	active

International Journal of
Molecular Sciences

Table S6. Structural conformer with the best **MPMERβ pharmacophore fit score**, identified in the virtual screening of the FDA and DrugBank databases.

Drug	Conformational-Fit	Matching pharmacophoric features	Pharmacophore-Fit Score
**CHEMBL193064**	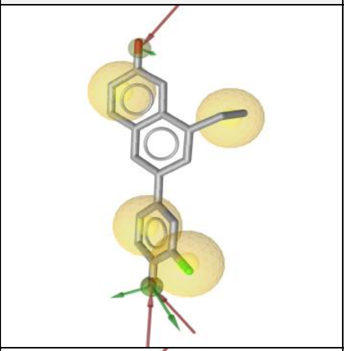	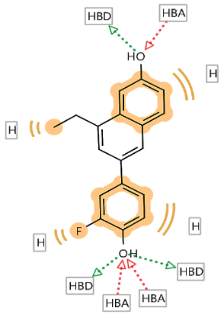	106.94
**DB06875**	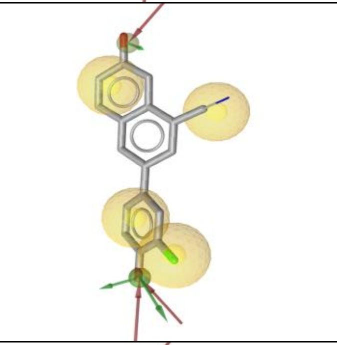	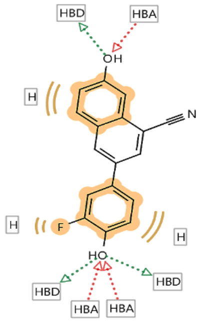	98.03
**S-DPN**	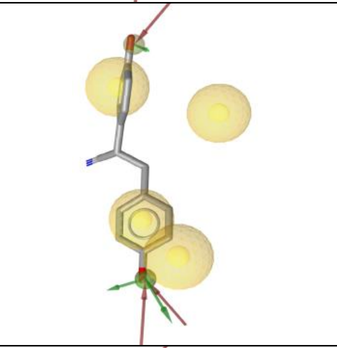	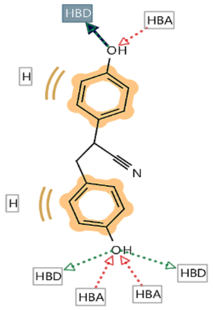	87.98
**DB06832**	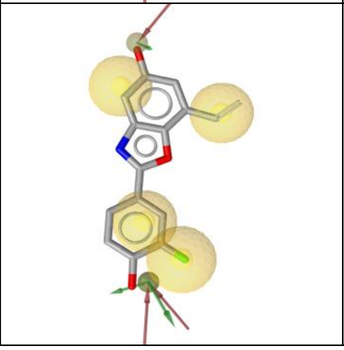	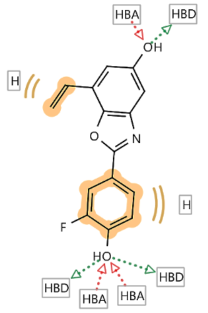	86.75

International Journal of Molecular Sciences

MDPI

**DB07425**	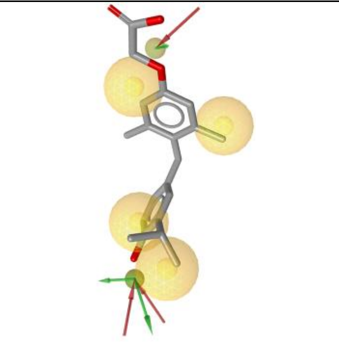	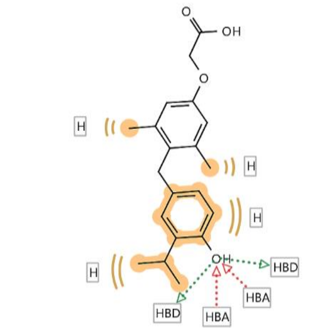	85.30
**DB01645**	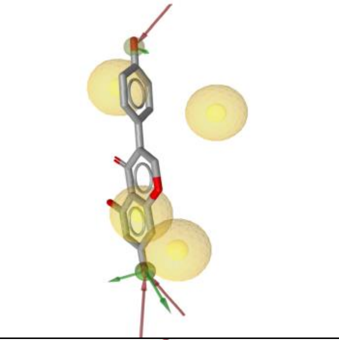	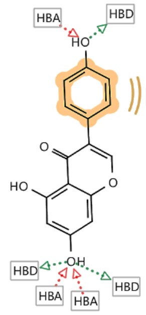	77.33
**Raloxifene**	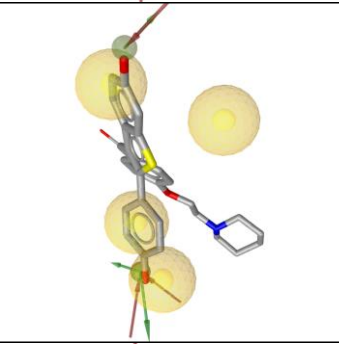	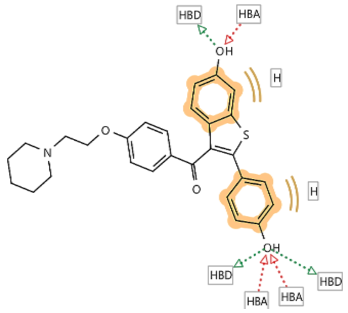	76.87
**Bazedoxifene**	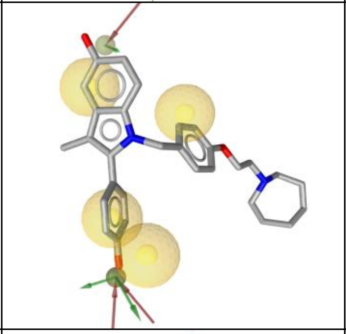	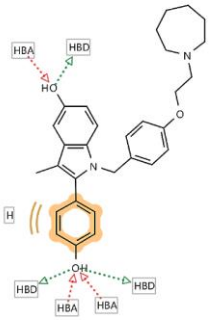	76.83
**Labetalol**	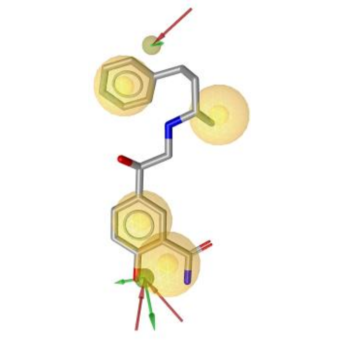	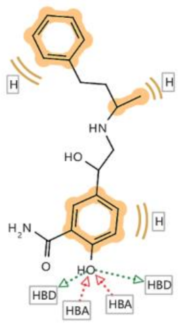	76.64

International Journal of
Molecular Sciences

**Procaterol**	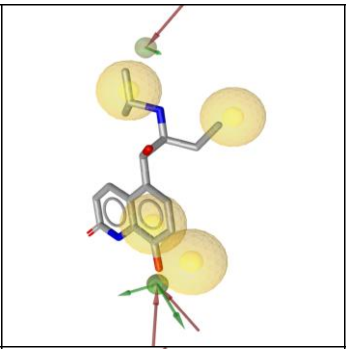	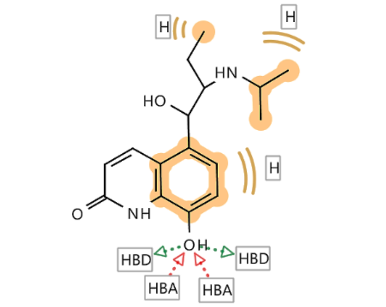	76.38
**GW368**	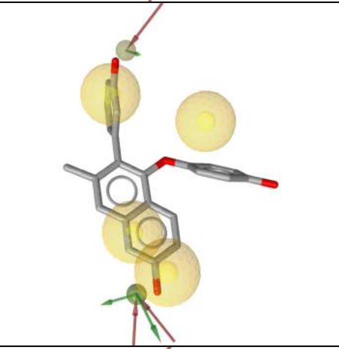	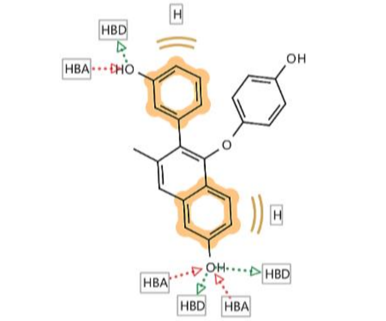	75.60
**DB00179**	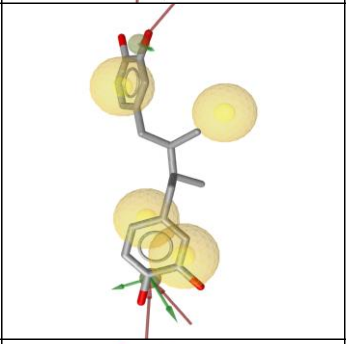	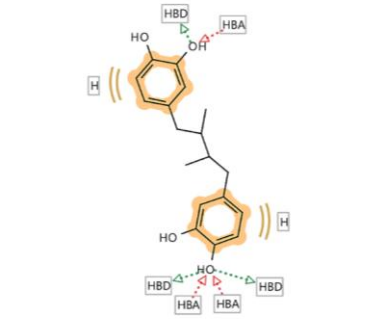	75.58
**Dobutamine**	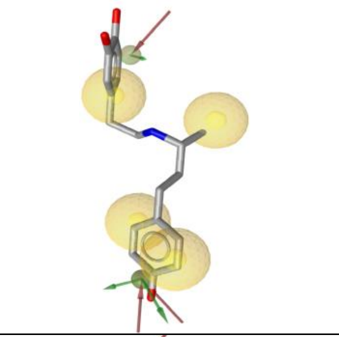	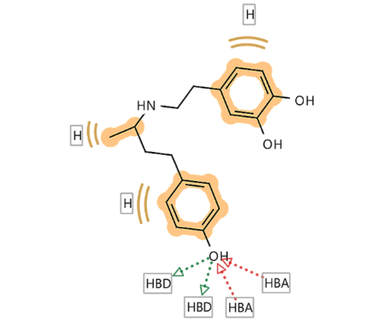	75.55
**DB15058**	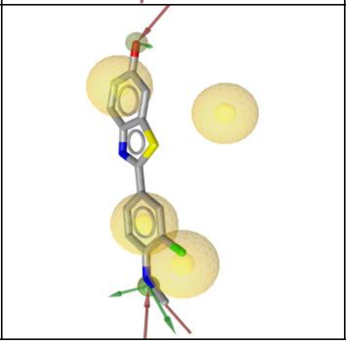	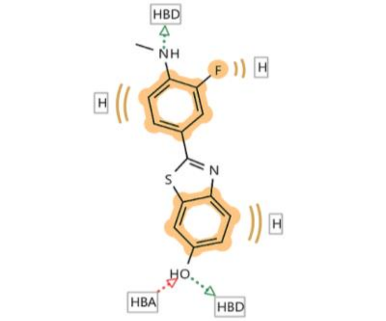	67.88

International Journal of Molecular Sciences

**Fenoldopam**	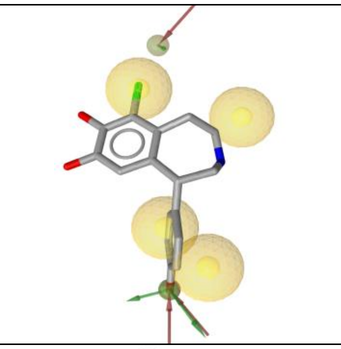	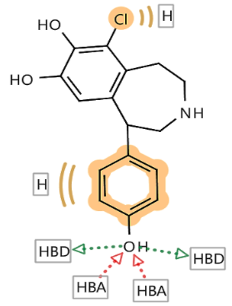	67.56
**Ritodrine**	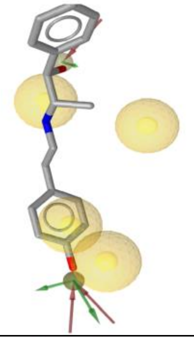	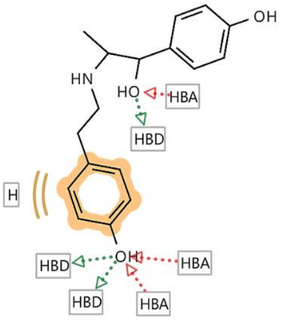	67.07
**DB14129**	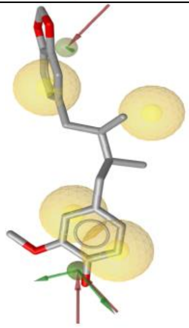	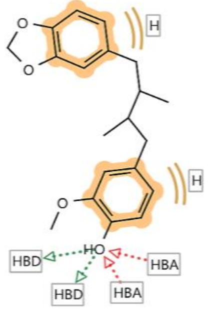	66.50
**DBMET00290**	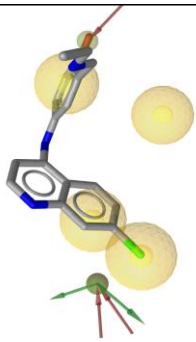	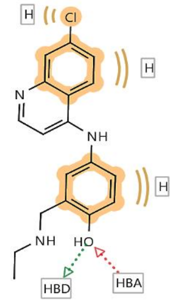	57.36

International Journal of
Molecular Sciences

MDPI

**Estradiol**	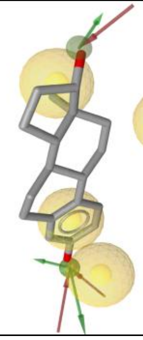	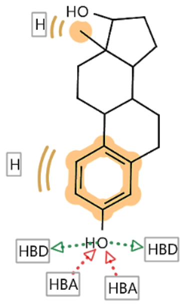	56.89
**Capsaicin**	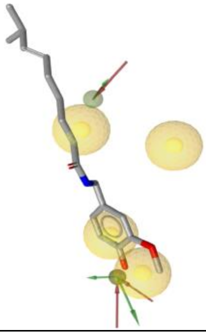	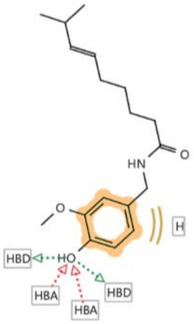	55.75
**Arbutamine**	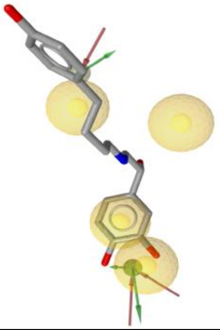	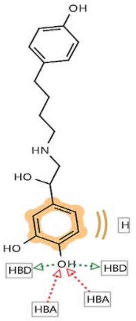	54.86
**Formoterol**	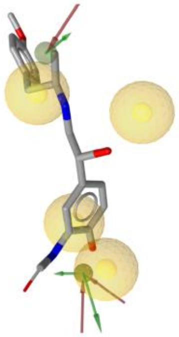	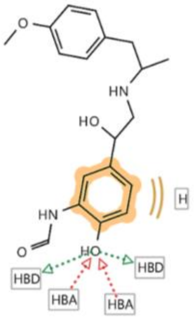	54.68
**Nadolol**	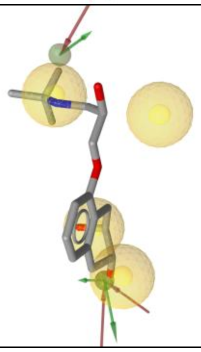	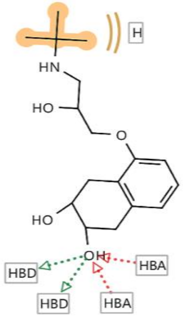	46.57

International Journal of Molecular Sciences

**Fulvestrant**	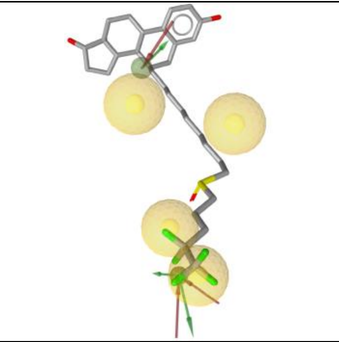	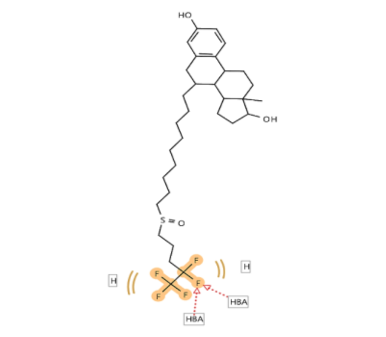	46.54
**Amodiaquine**	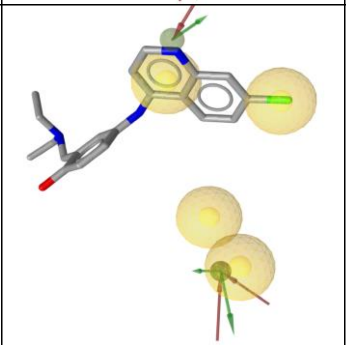	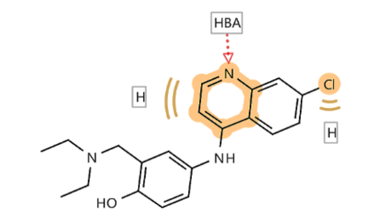	37.36
**DB04468**	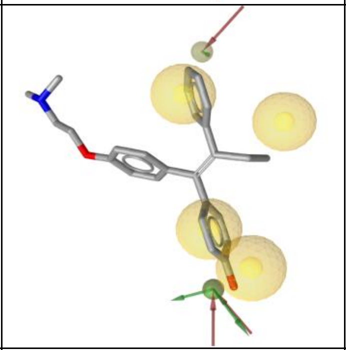	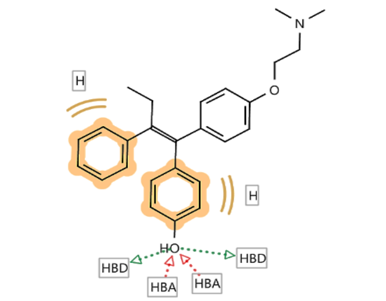	>35.0
**PPT**	undetermined	undetermined	>35.0
**Pravastatin**	undetermined	undetermined	>35.0
**Dipivefrin**	undetermined	undetermined	>35.0

International Journal of
Molecular Sciences

Table S7. RMSD values of the conformational overlap of the co-crystallized ligand (blue) and the theoretical conformer (olive green) obtained by molecular docking.

PDB ID	Ligand (co-crystallized)	RMSD (Å)	Superposition (RMSD-based)
1X7B	Prinaberel (ERβ agonist)	1.70	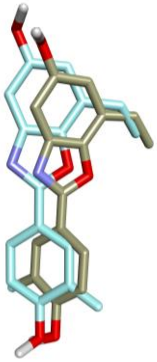
3DT3	GW368 (ERα agonist)	1.22	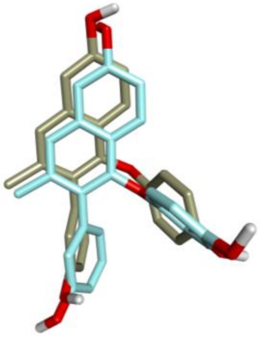

International Journal of Molecular Sciences

MDPI

Table S8. Interaction energy of drugs selected by virtual screening on ERβ, using molecular docking.

Drug	ΔG Blind Docking (kcal/mol)		ΔG Induced Docking (kcal/mol)		ERβ Interactions
	ERβ	ERα	ERβ	ERα	
**CHEMBL193064**	-11.4	-9.8	-10.38	-9.16	Leu298, Ala302, Glu305, Met336, Arg346, Phe356, Gly472, His475, Leu476, Leu491
**DB06875**	-11.1	-9.5	-10.29	-9.15	Leu298, Ala302, Glu305, Met336, Leu339, Arg346, Phe356, Gly472, His475, Leu476
**S-DPN**	-9.3	-8.6	-8.77	-8.24	Met295, Leu298, Glu305, Met336, Leu339, Met340, Leu343, Arg346, Phe356, Gly472, His475, Leu476
**DB06832**	-11	-9.9	-9.03	-8.27	Leu298, Ala302, Glu305, Met336, Leu339, Phe356, Gly472, His475, Leu476, Leu491
**DB07425**	-8.0	-8.2	-9.70	-8.44	Met295, Leu298, Leu301, Ala302, Glu305, Met336, Leu339, Met340, Leu343, Phe356, Gly472, His475, Leu476, Leu491
**DB01645**	-11.1	-10.1	-9.32	-8.35	Leu298, Leu301, Ala302, Glu305, Met336, Leu339, Leu343, Arg346, Phe356, Gly472, His475, Leu476
**Raloxifene**	--	-10.3	-8.86	-13.66	Met295, Leu298, Ala302, Glu305, Leu339, Met340, Leu343, Phe356, Ile373, Ile376, Leu380, Gly472, His475, Leu476
**Bazedoxifene**	--	-11.4	-8.88	-13.4	Met295, Leu298, Leu301, Ala302, Glu305, Met336, Leu339, Met340, Leu343, Phe356, Ile373, Ile376, Leu476
**Labetalol**	-8.6	-8.0	-9.51	-8.85	Leu298, Leu301, Ala302, Glu305, Met336, Leu339, Phe356, Ile373, Ile376
**Procaterol**	-8.5	-7.9	-8.71	-7.61	Leu298, Ala302, Glu305, Leu339, Leu343, Arg346, Phe356, Ile373, Ile376, Phe377, Leu380
**GW368**	--	-12.0	-9.91	-10.8	Met295, Leu298, Leu301, Ala302, Glu305, Trp335, Met336, Leu339, Met340, Phe356, Gly472, His475, Leu476, Leu491
**DB00179**	-9.1	-9.5	-9.56	-9.35	Leu298, Glu305, Met336, Leu339, Met340, Leu343, Arg346, Phe356, Leu380, Gly472, Leu476
**Dobutamine**	-9.4	-8.9	-8.87	-8.46	Met295, Leu298, Glu305, Leu339, Met340, Leu343, Arg346, Phe356, Gly472, Leu476
**DB15058**	-7.6	-6.6	-8.67	-7.72	Leu298, Ala302, Glu305, Met336, Leu339, Met340, Leu343, Arg346, Phe356, Gly472, Leu476
**Fenoldopam**	-9.0	-9.3	-8.14	-7.67	Met295, Leu298, Leu301, Ala302, Glu305, Met336, Leu339, Arg346, Ile373, His475
**Ritodrine**	-9.0	-8.7	-8.42	-8.37	Leu298, Glu305, Met336, Leu339, Leu343, Arg346, Gly472, His475, Leu476
**DB14129**	-8.8	-8.9	-10.40	-9.20	Leu298, Leu301, Ala302, Glu305, Trp335, Met336, Leu339, Phe356, Ile373, Leu476, Leu491
**DBMET00290**	-8.3	-8.4	-10.93	-9.80	Met295, Leu298, Glu305, Met336, Leu339, Met340, Leu343, Arg346, Phe356, Ile376, His475, Leu476
**Estradiol**	-11.7	-11.1	-10.74	-9.67	Met295, Leu298, Ala302, Glu305, Met336, Leu339, Leu343, Phe356, Ile373, Gly472, Leu476
**Capsaicin**	-7.7	-7.8	-8.96	-8.11	Leu298, Leu301, Ala302, Glu305, Met336, Leu339, Phe356, Ile373, Ile376, Phe377, Leu380
**Arbutamine**	-9.0	-8.2	-8.46	-7.94	Met295, Leu298, Ala302, Glu305, Met336, Leu339, Met340, Leu343, Arg346, Phe356, Leu476
**Formoterol**	-7.8	-8.0	-8.88	-8.27	Leu298, Glu305, Met336, Leu339, Met340, Leu343, Phe356, Leu476
**Nadolol**	-7.8	-8.4	-9.15	-8.15	Leu298, Glu305, Gly472, His475
**Fulvestrant**	--	-8.3	-1.91	-12.1	Leu298, Glu305, Met336, Leu339, Met340, Leu343, Arg346, Phe356, Ile373, Phe377, Leu380, Gly472
**Amodiaquine**	-5.9	-6.9	-9.92	-9.26	Met295, Leu298, Leu301, Ala302, Glu305, Leu339, Met340, Leu343, Arg346, Phe356, Ile376, His475, Leu476
**DB04468**	--	-8.6	-9.49	-10.7	Leu298, Ala302, Glu305, Trp335, Met336, Leu339, Met340, Leu343, Arg346, Phe356, Ile373, Ile376, Phe377, Leu380, Gly472, His475, Leu476, Leu491
**PPT**	--	-9.8	-10.06	-10.5	Leu301, Glu305, Trp335, Leu339, Arg346, Phe356, Leu491
**Pravastatin**	-7.3	-8.3	-10.39	-9.02	Leu298, Ala302, Glu305, Trp335, Met336, Leu339, Arg346, Phe356, Ile373, Ile376, Phe377, Leu380, His475, Leu476, Leu491
**Dipivefrin**	-7.1	-7.4	-9.93	-9.34	Met295, Leu298, Ala302, Glu305, Leu339, Phe356, Ile373, Ile376, Phe377, Leu380, His475, Leu476, Met479

-- It adopted a binding mode outside the active site

International Journal of
Molecular Sciences

MDPI

Table S8. …*continuation.* 2D diagram of molecular interactions of promising drugs within the ERβ active site.

CHEMBL193064	DB06875	S-DPN	DB06832
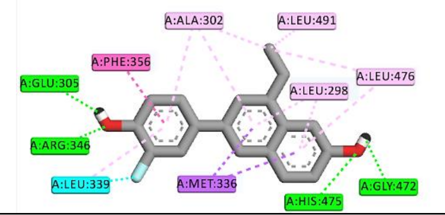	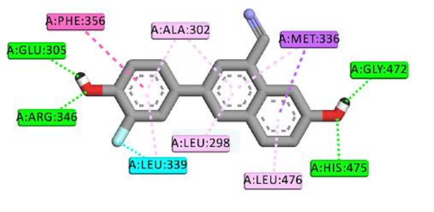	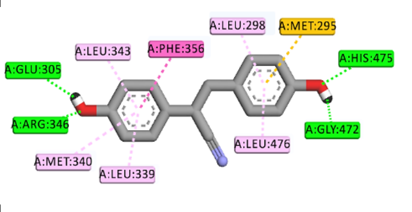	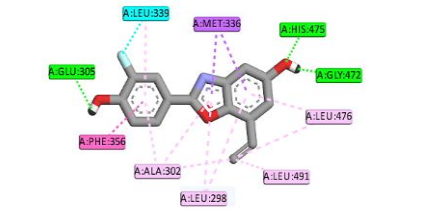
**DB07425**	**DB01645**	**Raloxifene**	**Bazedoxifene**
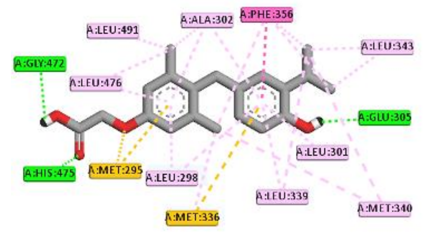	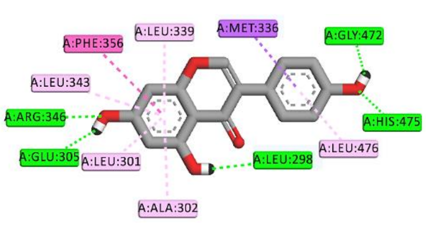	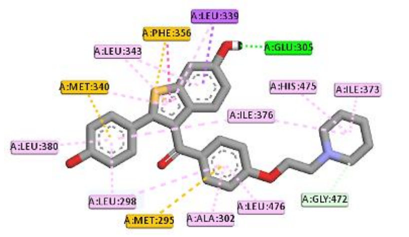	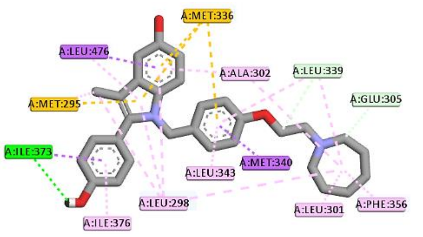
**Labetalol**	**Procaterol**	**GW368**	**DB00179**
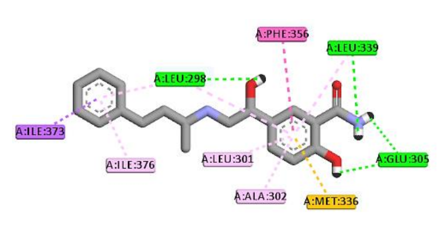	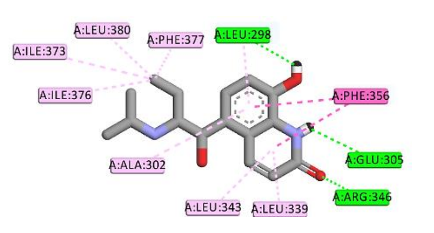	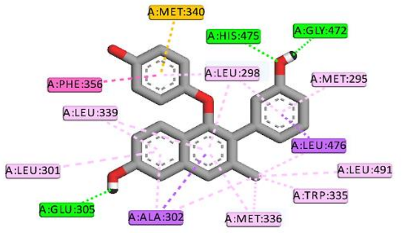	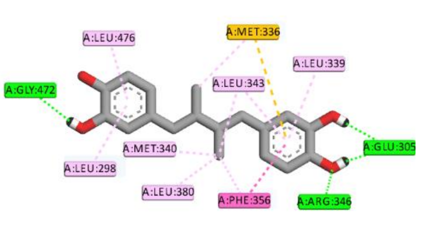
**Dobutamine**	**DB15058**	**Fenoldopam**	**Ritodrine**
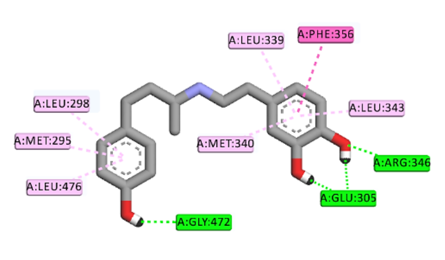	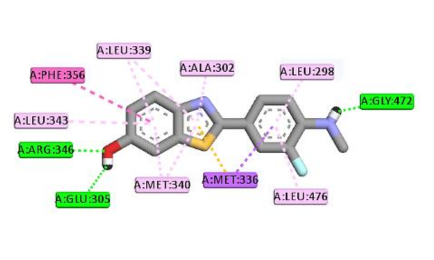	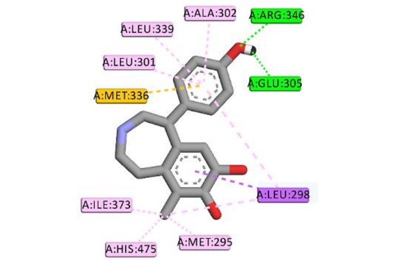	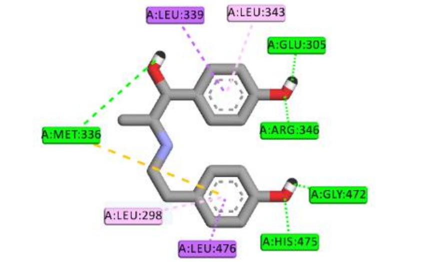

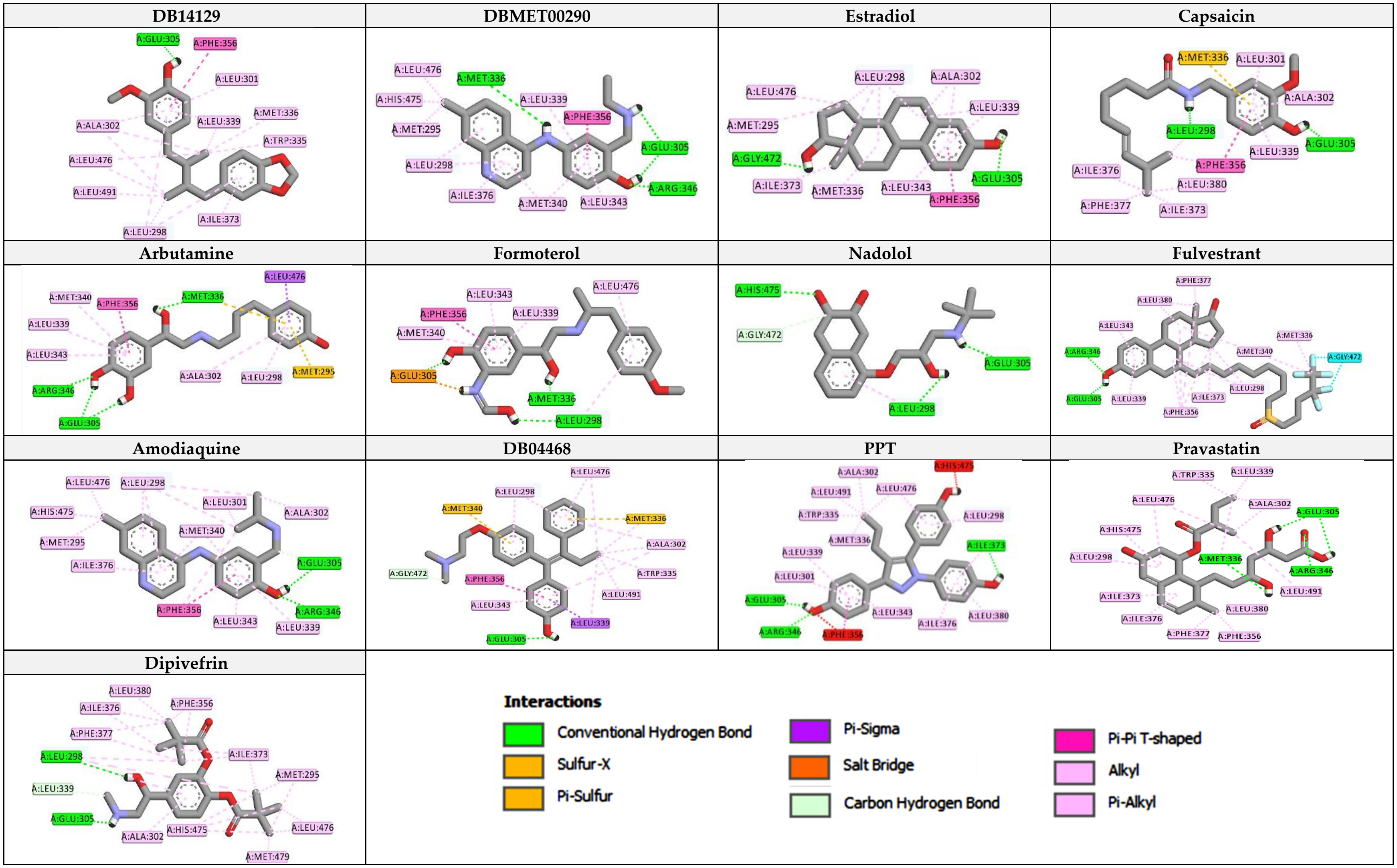
DB14129
DBMET00290
Estradiol
Capsaicin
Arbutamine
Formoterol
Nadolol
Fulvestrant
Amodiaquine
DB04468
PPT
Pravastatin
Dipivefrin
Interactions
Conventional Hydrogen Bond
Sulfur-X
Pi-Sulfur
Pi-Sigma
Salt Bridge
Carbon Hydrogen Bond
Pi-Pi T-shaped
Alkyl
Pi-Alkyl

International Journal of *Molecular Sciences*

MDPI

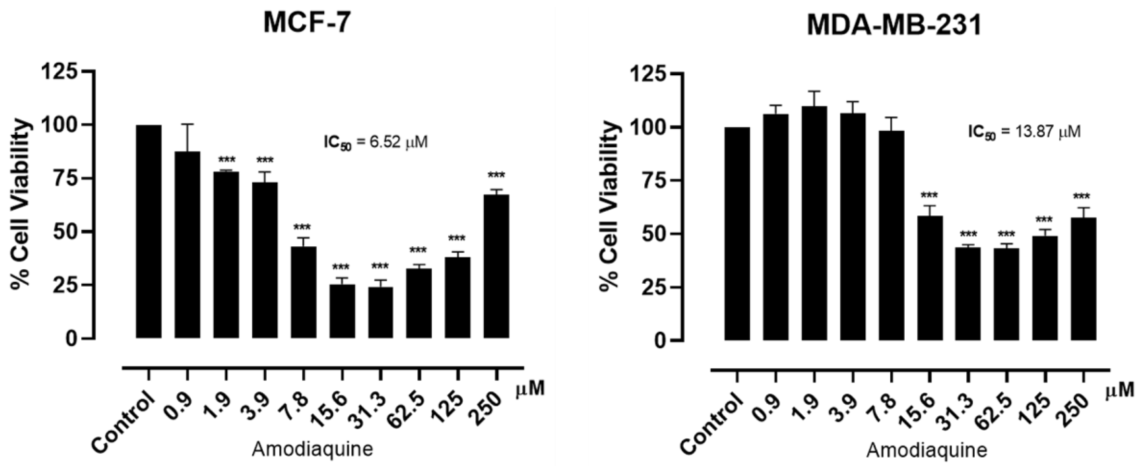

Figure S1. Antiproliferative activity of the Amodiaquine on the MCF-7 and MDA-MB-231 cell lines. MTT assay was used to determine the % of cell proliferation, the experiments were performed in triplicate with n = 6 for each concentration. The data are presented as means S.E. one-way ANOVA with Dunnett's post hoc test, *$p<0.05$, *$p<0.01$ and ***$p<0.001$ vs control.

References

104 Möcklinghoff, S.; Rose, R.; Carraz, M.; Visser, A.; Ottmann, C.; Brunsveld, L. Synthesis and crystal structure of a phosphorylated estrogen receptor ligand binding domain. *Chembiochem*. **2010**, *11*(16), 2251-4. doi: 10.1002/cbic.201000532.

105 Sun, W.; Cama, L.D.; Birzin, E.T.; et al. 6H-Benzo[c]chromen-6-one derivatives as selective ERbeta agonists. *Bioorg Med Chem Lett*. **2006**, *16*(6), 1468-1472. doi:10.1016/j.bmcl.2005.12.057.

106 Malamas, M.S; Manas, E.S.; McDevitt, R.E.; et al. Design and synthesis of aryl diphenolic azoles as potent and selective estrogen receptor-beta ligands. *J Med Chem*. **2004**, *47*(21), 5021-5040. doi:10.1021/jm049719y

107 McDevitt, R.E.; Malamas, M.S.; Manas, E.S.; Unwalla, R.J.; Xu, Z.B.; Miller. C.P.; Harris, H.A. Estrogen receptor ligands: design and synthesis of new 2-arylindene-1-ones. *Bioorg Med Chem Lett*. **2005**, *15*(12), 3137-3142. doi: 10.1016/j.bmcl.2005.04.013. PMID: 15876535.

108 Mewshaw, R.E.; Bowen, S.M.; Harris, H.A.; Xu, Z.B.; Manas, E.S.; Cohn, S.T. ERbeta ligands. Part 5: synthesis and structure-activity relationships of a series of 4'-hydroxyphenyl-aryl-carbaldehyde oxime derivatives. *Bioorg Med Chem Lett*. **2007**, *17*(4), 902-906. doi:10.1016/j.bmcl.2006.11.066

109 Richardson, T.I.; Dodge, J.A.; Wang, Y.; Durbin, J.D.; Krishnan, V.; Norman, B.H. Benzopyrans as selective estrogen receptor beta agonists (SERBAs). Part 5: Combined A- and C-ring structure-activity relationship studies. *Bioorg Med Chem Lett*. **2007**, *17*(20), 5563-5566. doi:10.1016/j.bmcl.2007.08.009

110 Manas, E.S.; Xu, Z.B.; Unwalla, R.J.; Somers, W.S. Understanding the selectivity of genistein for human estrogen receptor-beta using X-ray crystallography and computational methods. *Structure*. **2004**, 12(12), 2197-207. doi: 10.1016/j.str.2004.09.015.

111 Manas, E.S.; Unwalla, R.J.; Xu, Z.B.; Malamas, M.S.; Miller, C.P.; Harris, H.A.; Hsiao, C.; Akopian, T.; Hum, W.T.; Malakian, K.; Wolfrom, S.; Bapat, A.; Bhat, R.A.; Stahl, M.L.; Somers, W.S.; Alvarez, J.C. Structure-based design of estrogen receptor-beta selective ligands. *J Am Chem Soc*. **2004**, Nov 24;126(46):15106-19. doi: 10.1021/ja047633o.

112 Roberts, L.R.; Armor, D.; Barker, C.; *et al*. Sulfonamides as selective oestrogen receptor β agonists. *Bioorg Med Chem Lett*. **2011**, *21*(19), 5680-5683. doi:10.1016/j.bmcl.2011.08.041

113 Handa, C.; Yamazaki, Y.; Yonekubo, S.; Furuya, N.; Momose, T.; Ozawa, T.; Furuishi, T.; Fukuzawa, K.; Yonemochi, E.; Evaluating the correlation of binding affinities between isothermal titration calorimetry and fragment molecular orbital method of estrogen receptor beta with diarylpropionitrile (DPN) or DPN derivatives. *J Steroid Biochem Mol Biol*. **2022**, 222, 106152. doi: 10.1016/j.jsbmb.2022.106152.

114 Mewshaw, R.E.; Edsall, R.J. Jr.; Yang, C.; Manas, E.S.; Xu, Z.B.; Henderson, R.A.; Keith, J.C.Jr.; Harris, H.A. ERbeta ligands. 3. Exploiting two binding orientations of the 2-phenylnaphthalene scaffold to achieve ERbeta selectivity. *J Med Chem*. **2005**, *48*(12), 3953-79. doi: 10.1021/jm058173s

115 Wilkening, R.R.; Ratcliffe, R.W.; Tynebor, E.C.; Wildonger, K.J.; Fried, A.K.; Hammond, M.L.; Mosley, R.T.; Fitzgerald, P.M.; Sharma, N.; McKeever, B.M.; Nilsson, S.; Carlquist, M.; Thorsell, A.; Locco, L.; Katz, R.; Frisch, K.; Birzin, E.T.; Wilkinson, H.A.; Mitra, S.; Cai, S.; Hayes, E.C.; Schaeffer, J.M.; Rohrer, S.P. The discovery of tetrahydrofluorenones as a new class of estrogen receptor beta-subtype selective ligands. *Bioorg Med Chem Lett*. **2006**, *16*(13), 3489-94. doi: 10.1016/j.bmcl.2006.03.098.

116 Tanenbaum, D.M.; Wang, Y.; Williams, S.P.; Sigler, P.B. Crystallographic comparison of the estrogen and progesterone receptor's ligand binding domains. *Proc Natl Acad Sci USA*. **1998**, 95(11), 5998-6003. doi: 10.1073/pnas.95.11.5998.

117 Nettles, K.W.; Bruning, J.B.; Gil, G.; O'Neill, E.E.; Nowak, J.; Guo, Y.; Kim, Y.; DeSombre, E.R.; Dilis, R.; Hanson, R.N.; Joachimiak, A.; Greene, G.L. Structural plasticity in the oestrogen receptor ligand-binding domain. *EMBO Rep*. **2007**, *8*(6), 563-8. doi: 10.1038/sj.embor.7400963. Erratum in: *EMBO Rep*. **2007**, 8(6). 610. Guo, Yuee [added]; Hughs, Alun [removed]

118 Norman, B.H.; Richardson, T.I.; Dodge, J.A.; Pfeifer, L.A.; Durst, G.L.; Wang, Y.; Durbin, J.D.; Krishnan, V.; Dinn, S.R.; Liu, S.; Reilly, J.E.; Ryter, K.T. Benzopyrans as selective estrogen receptor beta agonists (SERBAs). Part 4: functionalization of the benzopyran A-ring. *Bioorg Med Chem Lett*. **2007**, *17*(18), 5082-5. doi: 10.1016/j.bmcl.2007.07.009

119 Fang, J.; Akwabi-Ameyaw, A.; Britton, J.E.; Katamreddy, S.R.; Navas, F. 3rd.; Miller, A.B.; Williams, S.P.; Gray, D.W.; Orband-Miller, L.A.; Shearin, J.; Heyer, D. Synthesis of 3-alkyl naphthalenes as novel estrogen receptor ligands. *Bioorg Med Chem Lett*. **2008**, *18*(18), 5075-7. doi: 10.1016/j.bmcl.2008.07.121

120 Delfosse, V.; Grimaldi, M.; Pons, J.L.; Boulahtouf, A.; le Maire, A.; Cavailles, V.; Labesse, G.; Bourguet, W.; Balaguer, P. Structural and mechanistic insights into bisphenols action provide guidelines for risk assessment and discovery of bisphenol A substitutes. *Proc Natl Acad Sci USA*. **2012**, *109*(37), 14930-5. doi: 10.1073/pnas.1203574109.

121 Osz, J.; Brélivet, Y.; Peluso-Iltis, C.; Cura, V.; Eiler, S.; Ruff, M.; Bourguet, W.; Rochel, N.; Moras, D. Structural basis for a molecular allosteric control mechanism of cofactor binding to nuclear receptors. *Proc Natl Acad Sci USA*. **2012**, *109*(10), E588-94. doi: 10.1073/pnas.1118192109.

122 Delfosse, V.; Grimaldi, M.; Cavaillès, V.; Balaguer, P.; Bourguet, W. Structural and functional profiling of environmental ligands for estrogen receptors. *Environ Health Perspect*. **2014**, *122*(12), 1306-13. doi: 10.1289/ehp.1408453

123 Delfosse, V.; Maire, A.L.; Balaguer, P.; Bourguet, W. A structural perspective on nuclear receptors as targets of environmental compounds. *Acta Pharmacol Sin*. **2015**, *36*(1), 88-101. doi: 10.1038/aps.2014.133

124 Nwachukwu, J.C.; Srinivasan, S.; Bruno, N.E.; Nowak, J.,Wright, N.J.; Minutolo, F.; Rangarajan, E.; Izard, T.; Yao, X.Q.; Grant, B.J.; Kojetin, D.J.; Elemento, O.; Katzenellenbogen; J.A.; Nettles, K.W. Systems Structural Biology Analysis of Ligand Effects on ER*α* Predicts Cellular Response to Environmental Estrogens and Anti-hormone Therapies. *Cell Chem Biol*. **2017**, 24(1), 35-45. doi: 10.1016/j.chembiol.2016.11.014
